# Regioselective Synthesis of New 2,4-(Het)aryl-3*H*-pyrido[1′,2′:1,5]pyrazolo[4,3-*d*]pyrimidines Involving Palladium-Catalyzed Cross-Coupling Reactions

**DOI:** 10.3390/molecules23112740

**Published:** 2018-10-23

**Authors:** Abdelaziz Ejjoummany, Rabia Belaroussi, Ahmed El Hakmaoui, Mohamed Akssira, Gérald Guillaumet, Frédéric Buron, Sylvain Routier

**Affiliations:** 1 Institut de Chimie Organique et Analytique, Univ Orleans, UMR CNRS 7311, BP 6759, F-45067 Orléans CEDEX 2, France; abdelaziz.ejjoummany@univ-orleans.fr (A.E.); rabia_belaroussi@hotmail.com (R.B.); gerald.guillaumet@univ-orleans.fr (G.G.); frederic.buron@univ-orleans.fr (F.B.); 2 Laboratoire de Chimie Physique et Chimie Bioorganique, Université Hassan II-Casablanca, B. P. 146, 28800 Mohammedia, Morocco; a_elhakmaoui@yahoo.fr

**Keywords:** pyridopyrazolopyrimidine, pyBroP activation, palladium cross-coupling

## Abstract

The design of some novel di-(het)arylated-3*H*-pyrido[1′,2′:1,5]pyrazolo[4,3-*d*]pyrimidine derivatives is reported. The series was developed from 1-aminopyridinium iodide, which afforded the key intermediate bearing two thiomethyl and amide functions, each of them useful for palladium catalyzed cross coupling reactions by alkyl sulfur release and C-O activation, respectively. The two regioselective and successive cross-coupling reactions were first carried out in C-4 by in situ C-O activation and next in C-2 by a methylsulfur release. Process optimization furnished conditions leading to products in high yields. The scope and limitations of the methodologies were evaluated and the final compounds characterized.

## 1. Introduction

The exploration of chemical space is a key prior step in the discovery of biologically active molecules. This strategy in heterocyclic chemistry includes the design and the functionalization of polynitrogen structures which are chosen for their similarity with major biomarkers and may contain scaffolds such as indoles, imidazoles, pyrimidine or imidazopyrimidine as well as tricyclic folate cores [1,2,3]. In view of their potential for the design of bioactive molecules, a variety of polynitrogenated skeletons have received considerable attention due to the synthetic challenge that they represent [4]. In this area, tricyclic fused heteroaromatic derivatives with a bridgehead nitrogen have less often been examined [5,6]. There is therefore a need to provide synthetic methods for their functionalization through reproducible and versatile strategies.

For several years, our group has been developing efficient methodologies to selectively functionalize heterocycles such as azaindoles [7,8,9], pyridopyrimidines [10,11,12,13,14,15,16], and more recently, tricyclic heterocycles containing a bridgehead nitrogen, such as pyrido[1′,2′:1,5]pyrazolo[3,4-*d*] pyrimidines or pyridazines [17,18,19,20,21].

**Figure 1:**
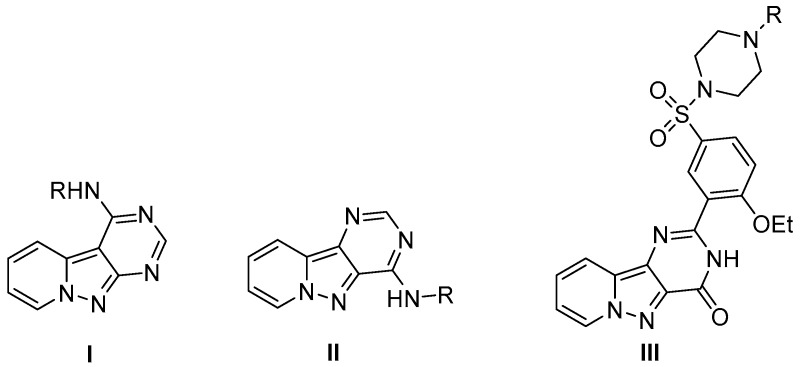
Some representative pyrido[1′,2′:1,5]pyrazolo[3,4-*d*]pyrimidine derivatives **I** and pyrido[1′,2′:1,5]pyrazolo[4,3-*d*]pyrimidine **II**, **III**.

Apart from the more classical pyrimidine ring, the currently most promising scaffold appears to be one containing a pyrazolopyridine, which has been used in diverse biologically and pharmaceutically active molecules including anticancer drugs [22], diuretic [23] and antiherpetic [24] drugs, and p38 kinase inhibitors [25]. With a view to increasing molecular diversity, tricyclic pyrido[1′,2′:1,5]pyrazolo[3,4-*d*]pyrimidines have emerged to provide high potential bioactive compounds (type **I**) [24]. Surprisingly, one isomer, the pyrido[1′,2′:1,5]pyrazolo[4,3-*d*]pyrimidine core, has seldom been described, and is reported in only three references, despite its significant growth potential as therapeutics for the central nervous system (type **II**, Figure 1) [26] or male erectile dysfunction treatment (type **III**) [27]. In order to design polyfunctionalized pyrido[1′,2′:1,5]pyrazolo[4,3-*d*]pyrimidines, we decided to explore its multiple substitution, an indispensable step to design future original bioactive molecules.

**Figure 2:**
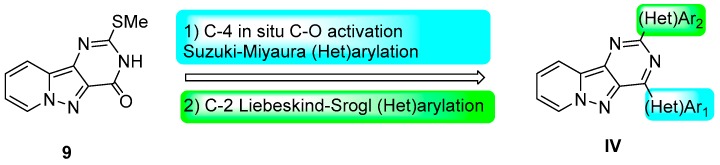
Bis(het)arylation of **9** leading to targeted structure **IV**.

In order to build C-2 and C-4 disubstituted pyrido[1′,2′:1,5]pyrazolo[4,3-*d*]pyrimidine derivatives, we developed a straightforward strategy which included from a versatile pattern **9**, a C-4 Suzuki-Miyaura cross coupling reaction using an in situ C-O activation [28,29] followed by a C-2 Liebeskind-Srogl cross-coupling reaction [20,30,31,32,33]. We report herein the preparation of **9** and its regioselective functionalization, the optimization of the experimental conditions and finally the scope of both reactions on these two identified positions (Figure 2).

## 2. Results and Discussion

**Scheme 1:**
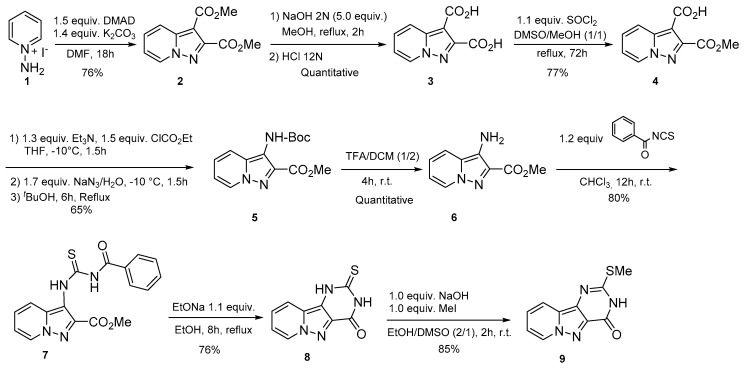
Synthetic pathway to generate **9**.

To design the platform **9**, we modified the available access (Scheme 1). Starting from the commercially available 1-aminopyridinium iodide (**1**), the 1,3-dipolar condensations of dimethyl acetylenedicarboxylate (DMAD) led to diester **2** in 78% yield (versus 29% in the literature [34]) and after saponification, the di-acid **3** was generated in a quantitative manner (64% in the literature). We next replaced the reported 4-step synthesis (decarboxylation, methylation, nitration, reduction) [27,34] by a shorter 3-step sequence. The discrimination of carboxylic acid functions was performed using an in situ anhydride formation followed by its regioselective opening with methanol to give the mono-methyl ester **4** [11]. This synthetic strategy allowed us to achieve a Curtius rearrangement on the residual acid function to produce, after cleavage of the Boc protective group, the hetaryl amine **6**. Finally, the condensation of benzoyl isothiocyanate with **6** followed by cyclization furnished **8** in basic media which was converted to the thiomethyl derivative **9** in satisfactory yield.

In order to tackle the usefulness of **9** as a building block and taking advantage of the presence of the amide group, we began by the regioselective functionalization of the C-4 position using a C-O direct activation involving PyBroP and Et_3_N to generate the required in situ generated *O*-phosphonium leaving group [35,36]. The second step required the addition of boronic acid, base, water and catalyst source.

We used conditions exploited in our previous research, which involved Na_2_CO_3_ and PdCl_2_(dppf).CH_2_Cl_2_ under thermal conditions. Under these conditions which have proved to be useful in diverse heterocyclic series, the reaction was successfully achieved with *para*-tolylboronic acid to afford **10** in a good 85% yield (Table 1, entry 1).

To evaluate the effect of boronic acid substituents, we first studied the reaction using other electron-donating groups such as methoxy in *ortho*, *meta* or *para* positions (entries 2–4). In *para* position, no alteration in the reaction efficiency was observed and compound **11** was isolated in 87% yield (entry 2). In contrast, the *ortho* orientation of the OMe group induced steric hindrance and consequently, a dramatic decrease in yield was observed. An intermediate yield was obtained with the *meta*-oriented methyl ester boronic acid (entries 3, 4) as the *meta*-methoxy group induces an inductive withdrawing effect. With an electron-withdrawing substituent on the phenylboronic acid, efficiency depends on the nature and strength of the electronic effects. With a moderate inductive effect, reactivity was maintained and **14** was isolated in a 90% yield. In the presence of strong electron- withdrawing substituents such as CF_3_ or CN the yields decreased to 70% and 58%, respectively (entries 6, 7). The same behaviour was observed with heteroaryl boronic acids. Electron-rich heterocycles such as 2-furan were introduced in good yield (74%, entry 9) whereas no reaction occurred with π-deficient heterocycles such as 4-pyridylboronic acid (entry 10). Finally, we investigated the interference of the OH group in basic media, which led to a moderate yield compared to its methyl ether equivalent (entry 8 vs. 2). In conclusion, the method is suitable with a wide variety of boronic acids, and limits concern only strongly deactivated (het)aryl boronic acids.

We next investigated the reactivity of the C-2 position. Methyl sulfur release was achieved using a Liebeskind-Srogl reaction which furnished the desired original 2,4-disubstituted compounds. The reactivity of the thioether **10** was explored using various (het)aryl boronic acids in the presence of Pd(PPh_3_)_4_ and copper(I) thiophene-2-carboxylate (CuTc) in THF at 100 °C under microwave irradiation (Table 2). As observed during the first cross coupling procedure, the use of electron-rich phenyl boronic acids was well tolerated and furnished the 4-tolyl- or 4-methoxyphenyl-substituted derivatives **20** and **21** in excellent yields (entries 1–2). The compartmental similarity between the two reactions is even clearer in the following results.

Firstly, the OMe position switch to the 2- or 3-methoxyphenyl boronic acids afforded the desired products in moderate yields (entries 3 and 4). Secondly, the presence of a strong electron-withdrawing CF_3_ group on the phenylboronic acid lowered the efficiency of the reaction. Nevertheless, the CF_3_ vs. F atom exchange fortunately restored the reactivity and led to **24** in a satisfactory 85% yield (entry 6 vs. 5) indicating that decreasing the electron-withdrawing character had a major impact on the reaction rate. This result was confirmed with (het)aryl boronic acids as electron-rich aromatics such as furan provided the desired product **27** in good yield whereas attempts with 4-pyridineboronic acid totally failed (entries 8,9).

To assess whether the nature of the C-4 residue really interferes with the C-2 reactivity, we varied the nature of 4-arylated tricyclic derivatives and fixed *p*-tolylboronic acid as sole arylation partner. When the pyrido[1′,2′:1,5]pyrazolo[4,3-*d*]pyrimidine derivative was substituted by a singly enriched 4-MeO-phenyl group at the C-4 position, the reaction gave derivative **29** in very good yield (entry 10), higher than that observed with **10** (entry 1). The inversion of the electronic effect in C-4 reduced the reactivity of the tricyclic system (entry 11) as was confirmed with the assay conducted starting from **14** that led to **30** in only 53% yield. In conclusion, the C-2 (het)arylation followed the same behavior as the C-4 cross coupling reaction with an additional favorable parameter i.e., the presence of electron rich (het)aryl moieties in C-4.

## 3. Materials and Methods

### 3.1. General Methods

Reactions were monitored by thin-layer chromatography (TLC) using silica gel (60 F254) plates, and the compounds were visualized by UV irradiation. Flash column chromatography was performed with silica gel 60 (230–400.13 mesh, 0.040 × 0.063 mm). The melting points were measured with samples in open capillary tubes. The infrared spectra of compounds were recorded with a Nicolet iS10 instrument (Thermo Scientific, Waltham, MA, USA). The ^1^H- and ^13^C-NMR spectra were recorded with DPX 250 (^13^C, 62 MHz), Avance II 250 (^13^C, 63 MHz), Avance 400 (^13^C, 101 MHz), or Avance III HD Nanobay 400 (^13^C, 101 MHz) spectrometers (Bruker, Billerica, MA, USA). The chemical shifts are given in ppm from tetramethylsilane as an internal standard. The coupling constants (*J*) are reported in Hz. High-resolution mass spectra (HRMS) were recorded with a Bruker Maxis 4G instrument Microwave irradiation was carried out in sealed vessels placed in a Initiator or Initiator + system (400 W maximum power, Biotage, Uppsala, Sweden). The temperatures were measured externally by IR. Pressure was measured by a noninvasive sensor integrated in the cavity lid.

### 3.2. Procedure to Synthesize Compounds ***2**–**18***, ***20**–**27*** and ***29***, ***30***

*Dimethyl pyrazolo[1,5-a]pyridine-2,3-dicarboxylate* (**2**) [34]: In a 100 mL flask, K_2_CO_3_ (0.87 g, 6.3 mmol, 1.4 equiv.) and DMAD (0.96 g, 6.8 mmol, 1.5 equiv.) were added to a solution of 1-aminopyridinium iodide (**1**, 1.0 g, 4.5 mmol) in anhydrous DMF (10 mL). The mixture was stirred at r.t. for 18 h. The solvent was evaporated in vacuo, then, the residue was purified by flash chromatography (EtOAc/petroleum ether, 2/8) to give **2** as yellow solid (744 mg, 76%). M.p. 64–66 °C; R*_f_* = 0.3 (EtOAc/petroleum ether, 3/7); IR (ATR diamond, cm^−1^): 3111, 1730, 1685, 1484, 1364, 1213, 1079, 995, 814; ^1^H-NMR (400 MHz, CDCl_3_): δ *=* 8.50 (d, *J* = 7.0 Hz, 1H), 8.15 (d, *J* = 7.5 Hz, 1H), 7.45 (t, *J* = 7.0 Hz, 1H), 7.02 (t, *J* = 7.5 Hz, 1H), 4.01 (s, 3H), 3.91 (s, 3H); ^13^C-NMR (101 MHz, CDCl_3_): δ = 163.2 (CO), 162.6 (CO), 147.3 (Cq), 141.4 (Cq), 129.2 (CH), 127.9 (CH), 119.9 (CH), 115.2 (CH), 102.7 (Cq), 53.0 (OCH_3_), 51.7 (OCH_3_) (^1^H-NMR and ^13^C-NMR of compounds **2**–1**8**, **20**–**27**, **29**, **30** are in Supplementary Materials); HRMS (ESI): *m*/*z* [M + H]^+^ calcd for C_11_H_11_N_2_O_4_: 235.0713; found: 235.0716.

*Pyrazolo[1,5-a]pyridine-2-dicarboxylic acid* (**3**) [34]: To a mixture of **2** (1.0 g, 4.3 mmol) in methanol (25 mL) was added a solution of NaOH 2 M in water (5.0 equiv.) and the mixture was refluxed for 1 h. The solvent was evaporated and the obtained residue was neutralized with an aqueous solution of 12 N HCl. After filtration, the precipitate obtained was washed with water (50 mL), and dried, to give **3** as a white solid (880 mg, quantitative). M.p. 216–218 °C; R*_f_* =0.1 (EtOAc/petroleum ether, 9/1); IR (ATR diamond, cm^−1^): 3475, 3341, 1697, 1506, 782; ^1^H-NMR (400 MHz, DMSO-*d*_6_): δ = 8.89 (d, *J* = 7.0 Hz, 1H), 8.17 (d, *J* = 7.0 Hz, 1H), 7.64 (t, *J* = 7.0 Hz, 1H). 7.26 (t, *J* = 7.0 Hz, 1H). ^13^C-NMR (101 MHz, DMSO-*d*_6_): δ = 164.7 (CO), 164.6 (CO), 148.2 (Cq), 141.5 (Cq), 130.4 (CH), 129.4 (CH), 119.7 (CH), 116.2 (CH), 102.5 (Cq); HRMS: *m*/*z* [M + H]^+^ calcd for C_9_H_7_N_2_O_4_: 207.0400, found: 207.0400.

2*-Methoxycarbonylpyrazolo[1,5-a]pyridine-3-carboxylic acid* (**4**): To a solution of **3** (0.5 g, 2.43 mmol) in DMSO/MeOH (1/1, 10 mL) was gradually added a SOCl_2_ solution (0.193 mL, 2.67 mmol, 1.1 equiv.) in methanol (10 mL) and the mixture was stirred at reflux for 72 h. The volatiles were evaporated and 15 mL of water were added. The resulting precipitate was filtered, then washed with water (3 × 25 mL) and dried to give **4** as a white solid (0.41 g, 77%). M.p. 214–216 °C; R*_f_* =0.3 (EtOAc/petroleum ether, 9/1); IR (ATR diamond, cm^−1^): 3012, 2744, 1730, 1440, 771; ^1^H-NMR (400 MHz, DMSO-*d*_6_): δ = 8.86 (d, *J* = 7.0 Hz, 1H), 8.10 (d, *J* = 7.0 Hz, 1H), 7.64 (t, *J* = 7.0 Hz, 1H), 7.24 (t, *J* = 7.0 Hz, 1H), 3.90 (s, 3H); ^13^C-NMR (101 MHz, DMSO-*d*_6_): δ = 164.2 (CO), 163.3 (CO), 147.9 (Cq), 140.8 (Cq), 130.3 (CH), 129.4 (CH), 119.3 (CH), 116.1 (CH), 102.4 (Cq), 53.3 (OCH_3_); HRMS: *m*/*z* [M + H]^+^ calcd for C_10_H_9_N_2_O_4_: 221.0553, found: 221.0556.

*Methyl 3-(tert-butoxycarbonylamino)pyrazolo[1,5-a]pyridine-2-carboxylate* (**5**): To a mixture of **4** (1.0 g, 4.5 mmol) in THF (15 mL) at −10 °C, was added triethylamine (597 mg, 0.82 mL, 5.9 mmol, 1.3 equiv.) and the mixture was stirred for 5 min. Ethyl chloroformate (739 mg, 6.8 mmol, 1.5 equiv.) was then added and the mixture was stirred for 1.5 h at −10 °C. A solution of NaN_3_ (0.501 g, 7.7 mmol, 1.7 equiv.) in water (2 mL) was added and the reaction mixture was stirred for 1.5 h at r.t. After filtration and evaporation, (15 mL) of water was added, and then the mixture was extracted with ethyl acetate (3 × 20 mL). After evaporation of the solvent, *tert*-butanol (20 mL) was added and the mixture was refluxed for 6 h. The reaction mixture was concentrated under reduced pressure. The product was purified by column flash chromatography (EtOAc/petroleum ether, 3/7); to give **5** as a white solid (0.85 g, 65%). M.p. 183–185 °C; R*_f_* = 0.5 (EtOAc/petroleum ether, 4/6); IR (ATR diamond, cm^−1^): 3412, 3090, 2975, 1542, 782; ^1^H-NMR (400 MHz, CDCl_3_): δ = 8.33 (d, *J* = 7.1 Hz, 1H), 8.13 (d, *J* = 7.1 Hz, 1H), 8.03 (s, NH), 7.05 (t, *J* = 7.1, 1H), 6.86 (t, *J* = 7.1Hz, 1H), 4.02 (s, 3H), 1.54 (s, 9H); ^13^C-NMR (101 MHz, CDCl_3:_) δ = 164.1 (CO), 153.0 (CO), 132.5 (Cq), 128.1 (CH), 121.8 (CH), 121.7 (CH), 116.3 (Cq), 114.8 (CH), 99.8 (Cq), 80.8 (CO), 52.2 (OCH_3_), 28.7 (3XCH_3_); HRMS: *m*/*z* [M + H]^+^ calcd for C_14_H_18_N_3_O_4_: 292.1293, found: 292.1291.

*Methyl 3-aminopyrazolo[1, 5-a]pyridine-2-carboxylate* (**6**): A solution of 3-*N*-Boc-aminoester **5** (0.5 g, 1.071 mmol) in a mixture of CH_2_Cl_2_/TFA (2/1) (15 mL) was stirred at r.t. for 4 h. After complete disappearance of the starting material, the volatiles were removed in vacuo then an aq. satd. solution of K_2_CO_3_ (10 mL) was slowly added. The residue was extracted with EtOAc (3 × 20 mL). The solvent was evaporated under reduced pressure and the crude material was purified by column chromatography (EtOAc/petroleum ether, 3/7) to give **6** as a yellow solid in quantitative manner. M.p. 186–184 °C, R*_f_* = 0.4 (EtOAc/petroleum ether, 5/5); IR (ATR diamond, cm^−1^): 3404, 3286, 1696, 1127, 910, 716; ^1^H-NMR (400 MHz, CDCl_3_): δ = 8.30 (d, *J* = 7.0 Hz, 1H), 7.44 (d, *J* = 7.0 Hz, 1H), 7.0 (t, *J* = 7.0 Hz, 1H), 6.80 (t, *J* = 7.0 Hz, 1H), 4.06 (s, 2H), 4.01 (s, 3H); ^13^C-NMR (101 MHz, CDCl_3_): δ = 164.6 (CO), 130.0 (Cq), 129.1 (Cq), 128.1 (CH), 126.0 (Cq), 119.8 (CH), 117.5 (CH), 114.8 (CH), 51.9 (OCH_3_); HRMS: *m*/*z* [M + H]^+^ calcd for C_9_H_10_N_3_O_2_: 192.0765, found: 192.0767.

*Methyl 3-(benzoylcarbamothioylamino)pyrazolo[1,5-a]pyridine-2-carboxylate* (**7**): To a solution of the amine **6** (85 mg, 0.44 mmol) in chloroform (5 mL) was added benzoyl isothiocyanate (87 mg, 0.53 mmol, 1.2 equiv.) and the mixture was stirred at r.t. for 12 h. After evaporating the solvent, the crude material was purified by column chromatography on silica gel, (EtOAc/petroleum ether, 2/8) to give the desired product **7** as a yellow solid (125 mg, 80%). M.p. 216–218 °C, IR (ATR diamond, cm^−1^): 3248, 3033, 1722, 1522, 1190, 750, 668; ^1^H-NMR (250 MHz, DMSO-*d*_6_): δ = 12.49 (s, NH), 11.79 (s, NH), 8.71 (d, *J* = 7.1 Hz, 1H), 8.00 (d, *J* = 8.0 H_Z_, 1H), 7.97 (d, *J* = 8.0 H_Z_, 1H), 7.68–7.60 (m, 2H), 7.52 (t, *J* = 7.1 Hz, 2H), 7.36–7.29 (m, 1H), 7.10 (t, *J* = 7.1 Hz, 1H), 3.83 (s, 3H); ^13^C-NMR (101 MHz, DMSO-*d*_6_): δ = 181.3 (CO), 172.6 (CS), 168.8 (CO), 162.3 (Cq), 137.9 (Cq), 135.8 (Cq), 133.7 (CH), 129.6 (CH), 129.2 (2 × CH), 129.0 (2 × CH), 124.7 (CH), 119.7 (CH), 115.7 (CH), 113.1 (Cq), 52.0 (OCH_3_); HRMS: *m*/*z* [M + H]^+^ calcd for C_17_H_15_N_4_O_3_S: 355.0862, found:355.0859.

*2-Thioxo-1H-pyrido[3,4]pyrazolo[4,3-d]pyrimidin-4-one* (**8**): To a solution of **7** (137 mg, 0.39 mmol) in ethanol (10 mL) was added a solution of sodium ethoxide (28.9 mg, 0.425 mmol, 1.1 equiv.). The reaction mixture was refluxed for 8 h. After evaporating the solvent, a mixture of water/acetic acid (4/1) was added. The obtained precipitate was filtered, washed with diethyl ether (2 × 20 mL) and the product **8** was obtained as a white solid (65 mg, 76%). M.p. 314–316 °C; IR (ATR diamond, cm^−1^): 3212, 2920, 1586, 1362, 839, 785; ^1^H-NMR (400 MHz, DMSO-*d*_6_): δ = 13.26 (s, NH), 12.40 (s, NH), 8.80 (d, *J* = 7.1 Hz, 1H), 8.16 (d, *J* = 7.1 Hz, 1H), 7.40 (t, *J* = 7.1 Hz, 1H), 7.23 (t, *J* = 7.1 Hz, 1H); ^13^C-NMR (101 MHz, DMSO-*d*_6_): δ = 172.9 (CO), 156.1 (CS), 132.9 (Cq), 129.6 (CH), 127.4 (Cq), 124.3 (CH), 120.8 (Cq), 119.1 (CH), 117.5 (CH); HRMS: *m*/*z* [M + H]^+^ calcd for C_9_H_7_N_4_OS: 219.0332, found: 219.0335.

*2-Methylsulfanyl-3H-pyrido[3,4]pyrazolo[4,3-d]pyrimidin-4-one* (**9**): To a solution of **8** (0.51 g, 2.33 mmol) in 10 mL of DMSO and 20 mL of ethanol was added NaOH (93 mg, 2.33 mmol, 1.0 equiv.). Iodomethane was slowly added (0.143 mL, 2.33 mmol, 1.0 equiv.) and the reaction was stirred at r.t. during 2 h. Then, the volatiles were evaporated and water (20 mL) was added. The resulting precipitate was filtered, washed with water (2 × 20 mL) and dried to give **9** as a yellow solid (270 mg, 85%). M.p. 302–304 °C; IR (ATR diamond, cm^−1^): 3232, 2927, 1596, 1365, 859, 775, ^1^H-NMR (250 MHz, DMSO-*d*_6_): δ = 12.52 (s, 1H), 8.88 (d, *J* = 7.0 Hz, 1H), 8.07 (d, *J* = 7.0 Hz, 1H), 7.43 (t, *J* = 7.0 Hz, 1H), 7.30 (t, *J* = 7.0 Hz, 1H), 2.61 (s, 3H); ^13^C-NMR (101 MHz, DMSO-*d*_6_): δ = 157.1 (CO), 152.7 (Cq), 135.2 (Cq), 133.5 (Cq), 130.6 (Cq), 129.8 (CH), 124.4 (CH), 118.6 (CH), 117.7 (CH), 13.5 (SCH_3_); HRMS: *m*/*z* [M + H]^+^ calcd for C_10_H_9_N_4_OS: 233.0489, found: 233.0491.

*General procedure A for the direct arylation via C-OH bond activation*: In a sealed tube of 10 mL, a solution of **9** (100 mg, 0.43 mmol) in dioxane (4 mL) was degassed by argon bubbling for 10 min, then PyBroP (240 mg, 0.51 mmol, 1.2 equiv.) and Et_3_N (1.29 mmol, 0.17 mL, 3.0 equiv.) were added successively. After 18 h at 80 °C, the reaction mixture was cooled and a solution containing the required (Het)arylboronic acid (2.0 equiv.), Na_2_CO_3_ (5.0 equiv.) and PdCl_2_(dppf).CH_2_Cl_2_ (10 mol%) in H_2_O (1 mL) was added. The sealed tube was stirred and heated at 110 °C for 24 h. After cooling at r.t. the volatiles were evaporated under reduced pressure and the crude material diluted in CH_2_Cl_2_ (20 mL). The organic layers were washed with water (10 mL), dried over MgSO_4_, filtered, and concentrated under vacuum. The crude mixture was purified by column chromatography on silica gel to give the desired mono-arylated compounds.

*2-Methylsulfanyl-4-(p-tolyl)pyrido[3,4]pyrazolo[4,3-d]pyrimidine* (**10**): Using *p*-tolylboronic acid as coupling reagent and following the general procedure A, the crude product was purified by flash chromatography on silica gel (EtOAc/petroleum ether, 0.5/9.5) to afford **10** as a yellow solid in a 85% yield. M.p. 206–208 °C; R*_f_* = 0.5 (EtOAc/petroleum ether, 0.5/9.5); IR (ATR diamond, cm^−1^): 2922, 1635, 1577, 1528, 1435, 1240, 1184, 1002, 799, 752, 634; ^1^H-NMR (400 MHz, CDCl_3_): δ = 8.85–8.79 (m, 3H), 8.38 (d, *J* = 7.0 Hz, 1H), 7.44–7.37 (m, 3H), 7.32 (t, *J* = 7.0 Hz, 1H), 2.78 (s, 3H), 2.47 (s, 3H); ^13^C-NMR (101 MHz, CDCl_3_): δ = 162.5 (Cq), 154.7 (Cq), 142.1 (Cq), 138.4 (Cq), 136.9 (Cq), 133.5 (Cq), 133.3 (Cq), 130.3 (2 × CH), 129.5 (2 × CH), 128.9 (CH), 123.0 (CH), 119.7 (CH), 119.2 (CH), 21.8 (CH_3_), 14.9 (SCH_3_); HRMS: *m*/*z* [M + H]^+^ calculated for C_17_H_15_N_4_S: 307.1013, found: 307.1011.

*4-(4-Methoxyphenyl)-2-methylsulfanyl-pyrido[3,4]pyrazolo[4,3-d]pyrimidine* (**11**): Using 4-methoxy-phenylboronic acid as coupling reagent and following the general procedure A, the crude product was purified by flash chromatography on silica gel (EtOAc/petroleum ether, 0.5/9.5) to afford **11** as a yellow solid in a 87% yield. M.p. 188–190 °C; R*_f_* = 0.5 (EtOAc/petroleum ether, 0.5/9.5); IR (ATR diamond, cm^−1^): 2924, 2866, 1728, 1433, 1272, 1122, 844, 635; ^1^H-NMR (250 MHz, CDCl_3_): δ = 8.93 (d, *J* = 6.7 Hz, 2H), 8.77 (d, *J* = 6.7 Hz, 1H), 8.35 (d, *J* = 6.7 Hz, 1H), 7.3 (t, *J* = 6.7 Hz, 1H), 7.29 (t, *J* = 6.7 Hz, 1H), 7.08 (d, *J* = 6.7 Hz, 2H), 3.90 (s, 3H), 2.76 (s, 3H); ^13^C-NMR (101 MHz, CDCl_3_): δ = 162.5 (Cq), 162.3 (Cq), 154.1 (Cq), 138.1 (Cq), 136.7 (Cq), 133.3 (Cq), 132.0 (2 × CH), 128.7 (CH), 128.5 (Cq), 122.8 (CH), 119.6 (CH), 119.0 (CH), 114.1 (2 × CH), 55.4 (OCH_3_), 14.8 (SCH_3_); HRMS: *m*/*z* [M + H]^+^ calcd for C_17_H_15_N_4_OS: 323.0959, found: 323.0961.

*4-(3-Methoxyphenyl)-2-methylsulfanyl-pyrido[3,4]pyrazolo[4,3-d]pyrimidine* (**12**): Using *3*-methoxy-phenylboronic acid as coupling reagent and following the general procedure A, the crude product was purified by flash chromatography on silica gel (EtOAc/petroleum ether, 5/5) to afford **12** as a yellow solid in a 67% yield. M.p. 154–156 °C; R*_f_* = 0.4 (EtOAc/petroleum ether, 5/5); IR (ATR diamond, cm^−1^): 2919, 2851, 1737, 1635, 1495, 1132, 844, 628; ^1^H-NMR (250 MHz, CDCl_3_): δ = 8.82 (d, *J* = 6.9 Hz, 1H), 8.57 (d, *J* = 6.9 Hz, 1H), 8.51–8.47 (m, 1H), 8.40 (d, *J* = 6.9 Hz, 1H), 7.46 (dd, *J* = 15.2, 7.8 Hz, 2H), 7.37–7.31 (m, 1H), 7.12 (dd, *J* = 8.2, 2.9 Hz, 1H), 3.97 (s, 3H), 2.79 (s, 3H); ^13^C-NMR (101 MHz, CDCl_3_): δ = 162.3 (Cq), 159.9 (Cq), 154.2 (Cq), 138.6 (Cq), 137.3 (Cq), 136.7 (Cq), 133.3 (Cq), 129.7 (CH), 128.8 (CH), 123.1 (CH), 123.0 (CH), 119.6 (CH), 119.3 (CH), 117.7 (CH), 114.8 (CH), 55.5 (OCH_3_), 14.9 (SCH_3_); HRMS: *m*/*z* [M + H]^+^ calcd for C_17_H_15_N_4_OS: 323.0960, found: 323.0961.

*4-(2-Methoxyphenyl)-2-methylsulfanyl-pyrido[3,4]pyrazolo[4,3-d]pyrimidine* (**13**): Using 2-methoxy-phenylboronic acid as coupling reagent and following the general procedure A, the crude product was purified by flash chromatography on silica gel (EtOAc/petroleum ether, 4/6) to afford **13** as a yellow solid in a 20% yield. M.p. 194–196 °C; R*_f_* = 0.3 (EtOAc/petroleum ether, 4/6); IR (ATR diamond cm^−1^): 3077, 2920, 1600, 1431, 1157, 878, 636; ^1^H-NMR (250 MHz, CDCl_3_): δ = 8.75 (d, *J* = 7.0 Hz, 1H), 8.40 (d, *J* = 7.0 Hz, 1H), 7.74 (d, *J* = 7.0 Hz, 1H), 7.52 (t, *J* = 7.0 Hz, 1H), 7.42 (t, *J* = 7.0 Hz, 1H), 7.29 (t, *J* = 7.0 Hz, 1H), 7.19–7.11 (m, 2H), 3.85 (s, 3H), 2.77 (s, 3H); ^13^C-NMR (101 MHz, CDCl_3_): δ = 162.5 (Cq), 157.9 (Cq), 157.0 (Cq), 137.8 (Cq), 137.0 (Cq), 133.5 (Cq), 131.6 (CH), 131.4 (CH), 128.7 (CH), 125.5 (Cq), 122.6 (CH), 120.8 (CH), 119.5 (CH), 118.9 (CH), 112.2 (CH), 56.0 (OCH_3_), 14.8 (SCH_3_); HRMS: *m*/*z* [M + H]^+^ calcd for C_17_H_15_N_4_OS: 323.0959, found: 323.0961.

*4-(4-Fluorophenyl)-2-methylsulfanyl-pyrido[3,4]pyrazolo[4,3-d]pyrimidine* (**14**): Using 4-fluorophenyl-boronic acid as coupling reagent and following the general procedure A, the crude product was purified by flash chromatography on silica gel (EtOAc/petroleum ether, 1/9) to afford **14** as a yellow solid in a 90% yield. M.p. 140–142 °C; R*_f_* = 0.6 (EtOAc/petroleum ether, 1/9); IR (ATR diamond cm^−1^): 3095, 2920, 2849, 1635, 1556, 1420, 1210, 880, 749, 640, 524; ^1^H-NMR (250 MHz, CDCl_3_): δ = 8.97 (t, *J* = 6.8 Hz, 2H), 8.79 (d, *J* = 6.8 Hz, 1H), 8.39 (d, *J* = 6.8 Hz, 1H), 7.56–7.20 (m, 4H), 2.78 (s, 3H); ^13^C-NMR (101 MHz, CDCl_3_): δ = 164.9 (d, *J* = 254.5 Hz, Cq), 162.4 (Cq), 153.2 (Cq), 138.5 (Cq), 138.5 (Cq), 136.6 (Cq), 133.4 (Cq), 132.4 (d, *J* = 8.7 Hz, 2 × CH), 128.7 (CH), 123.1 (CH), 119.5 (d, *J* = 33.8 Hz, 2 × CH), 115.8 (CH), 115.6 (CH), 14.6 (SCH_3_); HRMS: *m*/*z* [M + H]^+^ calcd for C_16_H_12_FN_4_S: 311.0762, found: 311.0761.

*2-Methylsulfanyl-4-[4-(trifluoromethyl)phenyl]pyrido[3,4]pyrazolo[4,3-d]pyrimidine* (**15**): Using 4-trifluoromethylphenyl boronic acid as coupling reagent and following the general procedure A, the crude product was purified by flash chromatography on silica gel (EtOAc/petroleum ether, 0.5/9.5) to afford **15** as a yellow solid in a 70% yield. M.p. 184–186 °C; R*_f_* = 0.6 (EtOAc/petroleum ether, 0.5/9.5); IR (ATR diamond cm^−1^): 3076, 1643, 1541, 1466, 1255, 1230, 1086, 857, 753, 620; ^1^H-NMR (400 MHz, CDCl_3_): δ = 9.04 (d, *J* = 8.2 Hz, 2H), 8.82 (d, *J* = 6.9 Hz, 1H), 8.42 (t, *J* = 6.9 Hz, 1H), 7.84 (d, *J* = 8.2 Hz, 2H), 7.48 (t, *J* = 6.9 Hz, 1H), 7.38 (t, *J* = 6.9 Hz, 1H), 2.79 (s, 3H); ^13^C-NMR (101 MHz, CDCl_3_): δ = 162.4 (Cq), 152.6 (Cq), 139.1 (q, *J* = 1.4 Hz, Cq), 139.0 (Cq), 136.7 (Cq), 133.4 (Cq), 132.7 (q, *J* = 33.0 Hz, Cq), 132.5 (Cq), 130.4 (2 × CH), 128.8(CH), 125.4 (q, *J* = 3.7 Hz, 2 × CH), 123.4 (CH), 119.7 (CH), 119.6 (CH), 14.7 (SCH_3_); HRMS: *m*/*z* [M + H]^+^ calcd for C_17_H_12_F_3_N_4_S: 361.0730, found: 361.0729.

*4-(2-Methylsulfanylpyrido[3,4]pyrazolo[4,3-d]pyrimidin-4-yl)benzonitrile* (**16**): Using 4-cyanophenyl-boronic acid as coupling reagent and following the general procedure A, the crude product was purified by flash chromatography on silica gel (EtOAc/petroleum ether, 2/8) to afford **16** as a yellow solid in a 58% yield. M.p. 230–232 °C; R*_f_* = 0.76 (EtOAc/petroleum ether, 2/8); IR (ATR diamond cm^−1^): 2921, 2223, 1731, 1548, 1494, 1255, 1275, 1018, 877, 748, 631; ^1^H-NMR (400 MHz, CDCl_3_): δ = 9.10 (d, *J* = 8.5 Hz, 1H), 8.85 (d, *J* = 6.9 Hz, 1H), 8.46 (d, *J* = 6.9 Hz, 1H), 7.89 (d, *J* = 8.5 Hz, 1H), 7.54 (t, *J* = 6.9 Hz, 1H), 7.43 (t, *J* = 6.9 Hz, 1H), 2.81 (s, 3H); ^13^C-NMR (101 MHz, CDCl_3_): δ = 162.4 (Cq), 151.7 (Cq), 139.9 (Cq), 139.3 (Cq), 136.5 (Cq), 133.5(Cq), 132.3 (2 × CH), 130.5 (2 × CH), 128.8 (CH), 123.6 (CH), 119.9 (CH), 119.8 (CH), 118.7 (Cq), 114.4 (Cq), 14.8 (SCH_3_); HRMS: *m*/*z* [M + H]^+^ calcd for C_17_H_12_N_5_S: 318.0808, found: 318.0807.

*4-(2-Methylsulfanylpyrido[3,4]pyrazolo[4,3-d]pyrimidin-4-yl)phenol* (**17**): Using 4-hydroxyphenylboronic acid as coupling reagent and following the general procedure A, the crude product was purified by flash chromatography on silica gel (EtOAc/petroleum ether, 3/7) to afford **17** as a yellow solid in a 45% yield. M.p. 254–256 °C; R*_f_* = 0.4 (EtOAc/petroleum ether, 2/8); IR (ATR diamond cm^−1^): 3100, 2928, 2214, 1607, 1550, 1494, 1274, 1128, 1021, 877, 748, 631; ^1^H-NMR (250 MHz, CDCl_3_): δ = 9.08 (d, *J* = 8.6 Hz, 2H), 8.83 (d, *J* = 6.9 Hz, 1H), 8.44 (d, *J* = 6.9 Hz, 1H), 7.87 (d, *J* = 8.6 Hz, 1H ), 7.51 (t, *J* = 6.9 Hz, 1H), 7.42 (t, *J* = 6.9 Hz, 1H), 2.79 (s, 3H); ^13^C-NMR (101 MHz, CDCl_3_): δ = 162.5 (Cq), 151.8 (Cq), 139.9 (Cq), 136.5 (Cq), 133.9 (Cq), 132.3 (2 × CH), 130.5 (2 × CH), 128.8 (CH), 123.6 (CH), 119.9 (CH), 119.8 (CH), 118.7 (Cq), 114.6 (Cq), 14.8 (SCH_3_); HRMS: *m*/*z* [M + H]^+^ calcd for C_16_H_13_N_4_OS: 309.0805, measured: 309.0804.

*4-(2-Furyl)-2-methylsulfanyl-pyrido[3,4]pyrazolo[4,3-d]pyrimidine* (**18**): Using 2-furanylboronic acid as coupling reagent and following the general procedure A, the crude product was purified by flash chromatography on silica gel (EtOAc/petroleum ether, 4/6) to afford **18** as a green solid in a 74% yield. M.p. 190–192 °C; R*_f_* = 0.6 (EtOAc/petroleum ether, 3/7); IR (ATR diamond cm^−1^): 2917, 2849, 1737, 1523, 1497, 1233, 1165, 1018, 885, 747, 516; ^1^H-NMR (400 MHz, CDCl_3_): ^1^H-NMR (400 MHz, CDCl_3_): δ = 8.83 (d, *J* = 7.0 Hz, 1H), 8.38 (d, *J* = 7.0 Hz, 1H), 8.06 (d, *J* = 4.4 Hz, 1H), 7.85 (d, *J* = 2.8 Hz, 1H), 7.46 (t, *J* = 7.0 Hz, 1H), 7.35 (t, *J* = 7.0 Hz, 1H), 6.73 (dd, *J* = 4.4, 2.8 Hz, 1H), 2.78 (s, 3H); ^13^C-NMR (101 MHz, CDCl_3_): δ = 162.6 (Cq), 149.7 (Cq), 146.6 (CH), 145.2 (Cq), 137.7 (Cq), 134.4 (Cq), 133.6 (Cq), 128.9 (CH), 123.2 (CH), 119.5 (CH), 119.1 (2CH), 112.8 (CH), 14.7 (SCH_3_); HRMS**:** *m*/*z* [M + H]^+^ calcd for C_14_H_11_N_4_OS: 283.0649, found: 283.0648.

*General procedure B for C-2 Liebeskind–Srogl cross-coupling reaction:* Compound **10**, **11** or **14** (0.15 mmol), aryl boronic acid (1.5 equiv.), Cu-(I) thiophene-2-carboxylate (CuTc) (3.0 equiv.), and Pd(PPh_3_)_4_ (10 mol%) were dissolved in anhydrous THF (5 mL) under argon in a microwave sealed vial. The reaction mixture was irradiated under microwave at 100 °C for 1h30. After cooling, the solvent was evaporated under reduced pressure and the residue was diluted in an aq. satd. NaHCO_3_ solution (10 mL). The aqueous layer was extracted with CH_2_Cl_2_ (3 × 10 mL). The combined organic layers were washed with water (5 mL), dried with anhydrous MgSO_4_, filtered, and concentrated under reduced pressure. The residue was purified by flash chromatography on silica gel to afford the desired compounds.

*2,4-Bis(p-tolyl)pyrido[3,4]pyrazolo[4,3-d]pyrimidine* (**20**): Starting from **10**, using *p*-tolylboronic acid as coupling reagent and following the general procedure B, the crude product was purified by flash chromatography on silica gel (EtOAc/petroleum ether, 1/9) to afford **20** as a yellow solid in a 78% yield. M.p. 176–178 °C; R*_f_* = 0.7 (EtOAc/petroleum ether, 2/8); IR (ATR diamond, cm^−1^): 2922, 2851, 1704, 1524, 1495, 1134, 1017, 834, 746; ^1^H-NMR (400 MHz, CDCl_3_): δ = 8.94 (d, *J* = 7.9 Hz, 2H), 8.83 (d, *J* = 7.0 Hz, 1H), 8.60 (d, *J* = 7.9 Hz, 2H), 8.49 (d, *J* = 7.0 Hz, 1H), 7.48–7.38 (m, 3H), 7.37–7.29 (m, 3H), 2.47 (s, 3H), 2.43 (s, 3H); ^13^C-NMR (101 MHz, CDCl_3_): δ = 156.5 (Cq), 153.9 (Cq), 141.5 (Cq), 139.5 (Cq), 138.2 (Cq), 137.5 (Cq), 136.3 (Cq), 134.8 (Cq), 134.0 (Cq), 130.0 (2 × CH), 129.4 (2 × CH), 129.3 (2 × CH), 128.9 (CH), 128.0 (2 × CH), 123.2 (CH), 119.7 (CH), 119.2 (CH), 21.7 (CH_3_), 21.5 (CH_3_); HRMS: *m*/*z* [M + H]^+^ calcd for C_23_H_19_N_4_: 351.1525, found: 351.1524.

*2-(4-Methoxyphenyl)-4-(p-tolyl)pyrido[3,4]pyrazolo[4,3-d]pyrimidine* (**21**): Starting from **10**, using 4-methoxyphenylboronic acid as coupling reagent and following the general procedure B, the crude product was purified by flash chromatography on silica gel (EtOAc/petroleum ether, 1/9) to afford **21** as an orange solid in a 75% yield. M.p. 202–204 °C; R*_f_* = 0.6 (EtOAc/petroleum ether, 1.5/8.5); IR (ATR diamond, cm^−1^): 2921, 2851, 1608, 1524, 1495, 1234, 1024, 750; ^1^H-NMR (400 MHz, CDCl_3_): δ = 8.98 (d, *J* = 8.5 Hz, 2H), 8.88 (d, *J* = 7.0 Hz, 1H), 8.71 (d, *J* = 8.5 Hz, 2H), 8.53 (d, *J* = 7.0 Hz, 1H), 7.50 (t, *J* = 7.0 Hz, 1H), 7.46 (d, *J* = 8.5 Hz, 2H) 7.38 (t, *J* = 7.0 Hz, 1H), 7.10 (d, *J* = 8.5 Hz, 2H), 3.94 (s, 3H), 2.52 (s, 3H); ^13^C-NMR (101 MHz, CDCl_3_): δ = 161.0 (Cq), 156.4 (Cq), 154.0 (Cq), 141.5 (Cq), 138.2 (Cq), 137.4 (Cq), 134.7 (Cq), 134.0 (Cq), 131.9 (Cq), 130.0 (2 × CH), 129.6 (2 × CH), 129.4 (2 × CH), 128.9 (CH), 123.1 (CH), 119.7 (CH), 119.2 (CH), 113.9 (2 × CH), 55.4 (OCH_3_), 21.7 (CH_3_); HRMS: *m*/*z* [M + H]^+^ calcd for C_23_H_19_N_4_O: 367.1554, found: 367.1553.

*2-(3-Methoxyphenyl)-4-(p-tolyl)pyrido[3,4]pyrazolo[4,3-d]pyrimidine* (**22**): Starting from **10**, using 3-methoxyphenylboronic acid as coupling reagent and following the general procedure B, the crude product was purified by flash chromatography on silica gel (EtOAc/petroleum ether, 3/7) to afford **22** as a yellow solid in a 48% yield. M.p. 130–132 °C; R*_f_* = 0.5 (EtOAc/petroleum ether, 5/5); IR (ATR diamond, cm^−1^): 2922, 2801, 1722, 1584, 1486, 1240, 1036, 745; ^1^H-NMR (250 MHz, CDCl_3_): δ = 8.97 (d, *J* = 8.5 Hz, 2H), 8.87 (d, *J* = 7.0 Hz, 1H), 8.53 (d, *J* = 8.5 Hz, 1H), 8.35 (d, *J* = 8.5 Hz, 1H), 8.31 (s, 1H), 7.53–7.34 (m, 5H), 7.04 (d, *J* = 7.0 Hz, 1H), 3.99 (s, 3H), 2.50 (s, 3H); ^13^C-NMR (101 MHz, CDCl_3_): δ = 159.9 (Cq), 156.1 (Cq), 153.9 (Cq), 141.6 (Cq), 140.5 (Cq), 138.2 (Cq), 137.6 (Cq), 137.6 (Cq), 134.9 (Cq), 130.0 (2 × CH), 129.5 (CH), 129.4 (2 × CH), 129.0 (CH), 123.4 (CH), 120.7 (CH), 119.7 (CH), 119.3 (CH), 115.6 (CH), 113.1 (CH), 55.5 (OCH_3_), 21.7 (CH_3_); HRMS: *m*/*z* [M + H]^+^ calcd for C_23_H_19_N_4_O: 367.1554, found: 367.1553.

*2-(2-Methoxyphenyl)-4-(p-tolyl)pyrido[3,4]pyrazolo[4,3-d]pyrimidine* (**23**): Starting from **10**, using 3-methoxyphenylboronic acid as coupling reagent and following the general procedure B, the crude product was purified by flash chromatography on silica gel (EtOAc/petroleum ether, 4/6) to afford **23** as a yellow solid in a 25% yield. M.p. 160–162 °C; R*_f_* = 0.5 (EtOAc/petroleum ether, 6/4); IR (ATR diamond, cm^−1^): 3068, 2923, 2836, 2211, 1636, 1543, 1188, 935, 736; ^1^H-NMR (250 MHz, CDCl_3_): δ = 8.89 (d, *J* = 8.3 Hz, 3H), 8.56 (d, *J* = 8.3 Hz, 1H), 7.94 (d, *J* = 7.5 Hz, 1H), 7.52–7.35 (m, 5H), 7.17–7.08 (m, 2H), 3.93 (s, 3H), 2.47 (s, 3H); ^13^C-NMR (101 MHz, CDCl_3_): δ = 157.9 (Cq), 157.3 (Cq), 154.2 (Cq), 141.5 (Cq), 137.8 (Cq), 137.1 (Cq), 134.8 (Cq), 133.9 (Cq), 132.1 (Cq), 130.1 (CH), 130.1 (2 × CH), 129.9 (CH), 129.4 (2 × CH), 128.9 (CH), 123.3 (CH), 120.8 (CH), 119.8 (CH), 119.2 (CH), 112.5 (CH), 56.2 (OCH_3_), 21.7 (CH_3_); HRMS: *m*/*z* [M + H]^+^ calcd for C_23_H_19_N_4_O: 367.1554, found: 367.1553.

*2-(4-Fluorophenyl)-4-(p-tolyl)pyrido[3,4]pyrazolo[4,3-d]pyrimidine* (**24**): Starting from **10**, using 4-fluorophenylboronic acid as coupling reagent and following the general procedure B, the crude product was purified by flash chromatography on silica gel (EtOAc/petroleum ether, 1/9) to afford **24** as a yellow solid in a 85% yield. M.p. 156–158 °C; R*_f_* = 0.6 (EtOAc/petroleum ether, 1.5/8.5); IR (ATR diamond, cm^−1^): 3061, 2920, 1524, 1493, 1427, 1375, 1208, 1135, 748; ^1^H-NMR (400 MHz, CDCl_3_): δ = 8.94 (d, *J* = 8.2 Hz, 2H), 8.84 (d, *J* = 6.9 Hz, 1H), 8.71 (d, *J* = 8.2 Hz, 2H), 8.48 (d, *J* = 6.9 Hz, 1H), 7.48 (t, *J* = 6.9 Hz, 1H), 7.43 (d, *J* = 8.2 Hz, 2H), 7.34 (d, *J* = 6.9 Hz, 1H), 7.22 (d, *J* = 8.2 Hz, 2H), 2.50 (s, 3H); ^13^C-NMR (101 MHz, CDCl_3_): δ = 164.0 (d, *J* = 251.8 Hz, Cq), 155.5 (Cq), 153.9 (Cq), 141.7 (Cq), 138.1 (Cq), 137.4 (Cq), 135.2 (d, *J* = 2.9 Hz, Cq), 134.7 (Cq), 133.8 (Cq), 130.0 (d, *J* = 8.4 Hz, 2 × CH), 130.0 (2 × CH), 129.4 (2 × CH), 129.0 (CH), 123.3 (CH), 119.5 (d, *J* = 28.9 Hz, 2 × CH), 115.4 (CH), 115.2 (CH), 21.7 (CH_3_); HRMS: *m*/*z* [M + H]^+^ calcd for C_22_H_16_FN_4_: 355.1355, found: 355.1353.

*4-(p-Tolyl)-2-[4-(trifluoromethyl)phenyl]pyrido[3,4]pyrazolo[4,3-d]pyrimidine* (**25**): Starting from **10**, using 4-trifluoromethylphenylboronic acid as coupling reagent and following the general procedure B, compound was purified by flash chromatography on silica gel (EtOAc/petroleum ether, 1/9) to afford **25** as a yellow solid in a 60% yield. M.p. 174–176 °C; R*_f_* = 0.7 (EtOAc/petroleum ether, 2/8); IR (ATR diamond, cm^−1^): 2953, 2924, 2852, 1615, 1527, 1508, 1429, 1355, 1295, 1177, 797; 736; ^1^H-NMR (400 MHz, CDCl_3_): δ = 8.91 (d, *J* = 8.0 Hz, 2H), 8.83 (d, *J* = 7.0 Hz, 1H), 8.79 (d, *J* = 8.0 Hz, 2H), 8.46 (d, *J* = 7.0 Hz, 1H), 7.77 (d, *J* = 8.0 Hz, 2H), 7.49 (t, *J* = 7.0 Hz, 1H), 7.42 (d, *J* = 8.0 Hz, 2H), 7.36 (t, *J* = 7.0 Hz, 1H), 2.49 (s, 3H); ^13^C-NMR (101 MHz, CDCl_3_) δ = 154.6 (Cq), 153.8 (Cq), 142.3 (q, *J* = 1.3 Hz, Cq) 141.8 (Cq), 138.1 (Cq), 137.5 (Cq), 134.9 (Cq), 133.6 (Cq), 130.8 ( Cq), 130.0 (2 × CH), 129.3 (q, *J* = 251.3, CF_3_),129.4 (2 × CH), 129.0 (CH), 128.2 (2 × CH), 125.3 (q, *J* = 3.8 Hz, 2 × CH), 123.7 (CH), 119.6 (CH), 119.5 (CH), 21.7 (CH_3_); HRMS: *m*/*z* [M + H]^+^ calcd for C_23_H_16_F_3_N_4_: 405.1320, found: 405.1321.

*4-[4-(p-Tolyl)pyrido[3,4]pyrazolo[4,3-d]pyrimidin-2-yl]phenol* (**26**): Starting from **10**, using 4-hydroxyphenylboronic acid as coupling reagent and following the general procedure B, the crude product was purified by flash chromatography on silica gel (EtOAc/petroleum ether, 2.5/7.5) to afford **26** as a yellow solid in a 40% yield. M.p. 182–184 °C; R*_f_* = 0.4 (EtOAc/petroleum ether, 3/7); IR (ATR diamond, cm^−1^): 3108, 2923, 2215, 1615, 1547, 1508, 1429, 1355, 1293, 1157, 787, 746; ^1^H-NMR (250 MHz, Acetone-*d*_6_): δ = 9.08 (t, *J* = 7.8 Hz, 3H), 8.63 (d, *J* = 8.7 Hz, 2H), 8.54 (d, *J* = 7.8 Hz, 1H), 7.75–7.59 (m, 2H), 7.48 (d, *J* = 7.8 Hz, 2H), 7.02 (d, *J* = 8.7 Hz, 2H), 2.48 (s, 3H); ^13^C-NMR (101 MHz, Acetone-*d*_6_): δ = 159.2 (Cq), 155.9 (Cq), 153.0 (Cq), 141.6 (Cq), 138.2 (Cq), 136.9 (Cq), 134.5 (Cq), 134.1 (Cq), 130.6 (Cq), 129.9 (2 × CH), 129.5 (2 × CH), 129.3 (CH), 129.2 (2 × CH), 124.0 (CH), 120.0 (CH), 119.1 (CH), 115.2 (2 × CH), 20.7 (CH_3_); HRMS: *m*/*z* [M + H]^+^ calculated for C_22_H_17_N_4_O: 353.1397, found: 353.1396.

*2-(2-Furyl)-4-(p-tolyl)pyrido[3,4]pyrazolo[4,3-d]pyrimidine* (**27**): Starting from **10**, using 2-furanylboronic acid as coupling reagent and following the general procedure B, the crude product was purified by flash chromatography on silica gel (EtOAc/petroleum ether, 2/8) to afford **27** as a yellow solid in a 60% yield. M.p. 146–148 °C; R*_f_* = 0.6 (EtOAc/petroleum ether, 3/7); IR (ATR diamond, cm^−1^): 3542, 3100, 2922, 2852, 1715, 1525, 1508, 1356, 1275, 1182, 783, 753,612; ^1^H-NMR (400 MHz, CDCl_3_) δ 8.93 (d, *J* = 8.2 Hz, 2H), 8.88 (d, *J* = 6.9 Hz, 1H), 8.60 (d, *J* = 6.9 Hz, 1H), 7.71 (d, *J* = 3.6 Hz, 1H), 7.54–7.48 (m, 2H), 7.45 (d, *J* = 8.2 Hz, 2H), 7.39 (t, *J* = 6.9 Hz, 1H), 6.65 (dd, *J* = 3.6, 1.7 Hz, 1H), 2.51 (s, 3H); ^13^C-NMR (101 MHz, CDCl_3_): δ = 154.6 (Cq), 153.6 (Cq), 150.1 (Cq), 144.2 (CH), 141.8 (Cq), 137.4 (Cq), 137.3 (Cq), 134.6 (Cq), 133.4 (Cq), 130.0 (2 × CH), 129.4 (2 × CH), 128.9 (CH), 123.4 (CH), 119.8 (CH), 119.3 (CH), 112.1 (CH), 112.0 (CH), 21.7 (CH_3_); HRMS: *m*/*z* [M + H]^+^ calcd for C_20_H_15_N_4_O: 327.1241, found: 327.1240.

*2-(2-Tolyl)-4-(p-methoxyphenyl)pyrido[3,4]pyrazolo[4,3-d]pyrimidine* (**29**): Starting from **11**, using *p*-tolylboronic acid as coupling reagent and following the general procedure B, the crude product was purified by flash chromatography on silica gel (EtOAc/petroleum ether, 3/7) to afford **29** in a 86% yield. M.p. 210–212 °C; R*_f_* = 0.4 (EtOAc/petroleum ether, 3/7); IR (ATR diamond, cm^−1^): 3012, 1633, 1531, 1432, 1254, 1114, 1021, 804, 744; ^1^H-NMR (400 MHz, CDCl_3_): δ = 8.90 (d, *J* = 6.8 Hz, 1H), 8.74 (d, *J* = 8.8 Hz, 2H), 8.10 (d, *J* = 8.8 Hz, 1H), 7.94 (d, *J* = 8.8 Hz, 2H), 7.55–7.39 (m, 3H), 7.38–7.26 (m, 1H), 7.03 (d, *J* = 8.8 Hz, 2H), 3.90 (s, 3H), 2.52 (s, 3H) ppm; ^13^C-NMR (101 MHz, CDCl_3_): δ =164.4 (Cq), 163.9 (Cq), 162.4 (Cq), 141.6 (Cq), 136.4 (Cq), 136.1 (Cq), 131.8 (Cq), 131.3 (2 × CH), 130.2 (CH), 130.2 (2 × CH), 129.5 (2 × CH), 126.6 (CH), 120.3 (CH), 118.4 (CH), 114.3 (2 × CH), 103.3 (Cq), 91.0 (Cq), 56.0 (OCH_3_), 22.2 (CH_3_); HRMS: *m*/*z* [M + H]^+^ calcd for C_23_H_19_N_4_O [M + H]^+^ 367.1553, found 367.1556.

*2-(2-Tolyl)-4-(p-fluorophenyl)pyrido[3,4]pyrazolo[4,3-d]pyrimidine* (**30**): Starting from **14**, using *p*-tolyl-boronic acid as coupling reagent and following the general procedure B, the crude product was purified by flash chromatography on silica gel (EtOAc/petroleum ether, 2/8) to afford **30** in a 53% yield. M.p. 160–162 °C; R*_f_* = 0.6 (EtOAc/petroleum ether, 2/8); IR (ATR diamond, cm^−1^): 3061, 2920, 1524, 1493, 1427, 1375, 1208, 1135, 748; ^1^H-NMR (400 MHz, CDCl_3_) δ = 9.13 (d, *J* = 8.7, Hz, 2H), 8.87 (d, *J* = 6.9 Hz, 1H), 8.63 (d, *J* = 7.8 Hz, 2H), 8.54 (d, *J* = 6.9 Hz, 1H), 7.52 (t, *J* = 6.9 Hz, 1H), 7.43–7.30 (m, 5H), 2.49 (s, 3H); ^13^C-NMR (101 MHz, CDCl_3_) δ = 164.8 (d, *J* = 251.8 Hz, Cq), 156.5 (Cq), 152.5 (Cq), 139.6 (Cq), 138.4 (Cq), 137.3 (Cq), 136.1 (Cq), 134.8 (Cq), 132.9 (d, *J* = 3.0 Hz, Cq), 132.3 (d, *J* = 8.6 Hz, 2 × CH), 129.3 (2 × CH), 128.9 (CH), 127.9 (2 × CH), 123.4 (CH), 119.6 (d, *J* = 32.1 Hz, 2 × CH), 115.8 (CH), 115.5 (CH), 21.4 (CH_3_); HRMS: *m*/*z* [M + H]^+^ calcd for C_22_H_16_FN_4_: 355.1354, found: 355.1352.

## 4. Conclusions

In summary, we have described in this work a synthetic pathway for the preparation of an original pyrido[1′,2′:1,5]pyrazolo[4,3-*d*]pyrimidine platform, and then, have developed several arylations at its C-2 and C-4 positions. First, the amide function in C-4 position was reacted in a direct C–O activation with PyBroP as activator followed by a Suzuki-Miyaura cross coupling reaction to generate a library of C-4 (het)arylated derivatives and next a Liebeskind–Srogl reaction furnished the desired di-(het)arylated derivatives. The scope of the two reactions was investigated and showed a strong influence of electronic effect and steric hindrance. In both cases electron enrichment of the systems, i.e., the tricyclic core as well as the boronic partner, improved efficiency. This route will offer the opportunity to explore other metal catalyzed cross coupling reactions and to open a new chemical space area to generate new bioactive compounds containing polyfunctionalized pyrido[1′,2′:1,5]pyrazolo[4,3-*d*]pyrimidines.

## Figures and Tables

**Table 1:**
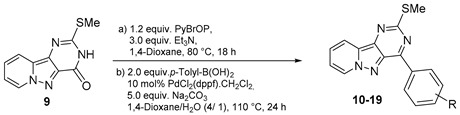
Scope of (het)arylation of **9** under C-O amide activation.

Entry	Product		Yield ^a^	Entry	Product		Yield ^a^
1	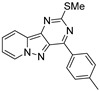	**10**	85	6	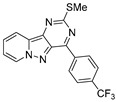	**15**	70
2	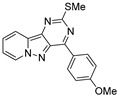	**11**	87	7	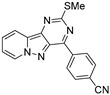	**16**	58
3	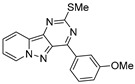	**12**	67	8	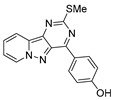	**17**	45
4	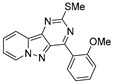	**13**	20	9	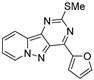	**18**	74
5	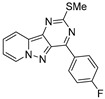	**14**	90	10	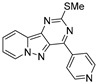	**19**	traces

^a^ Yield is indicated as isolated compound.

**Table 2:**
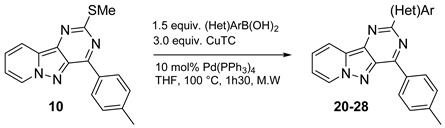
Scope of the desulfurative cross coupling reaction in C-2.

Entry	Product		Yield ^a^	Entry	Product		Yield ^a^
1	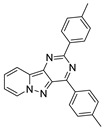	**20**	78	7	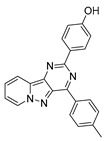	**26**	40
2	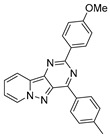	**21**	75	8	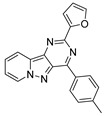	**27**	60
3	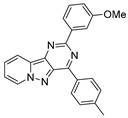	**22**	48	9	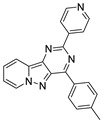	**28**	ND
4	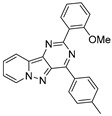	**23**	25	10	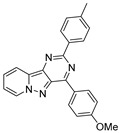	**29**	86
5	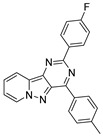	**24**	85	11	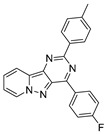	**30**	53
6	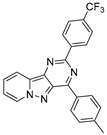	**25**	60				

^a^ Yield is indicated as isolated compound. ND: Not Detected.

## Supplementary Materials

The following are available online: ^1^H-NMR and ^13^C-NMR of compounds **2**–1**8**, **20**–**27**, **29**, **30**.
