# Supplementary material for: Regioselective Synthesis of New 2,4-(Het)aryl-3H-pyrido[1′,2′:1,5]pyrazolo[4,3-d]pyrimidines Involving Palladium-Catalyzed Cross-Coupling Reactions
Source: Molecules. 2018 Oct 23;23(11):2740. doi: 10.3390/molecules23112740 (PMC6278517; doi:10.3390/molecules23112740)

molecules

MDPI

Regioselective synthesis of new 2,4-(het)aryl-3*H*-pyrido[1',2':1,5]pyrazolo[4,3-*d*]pyrimidine involving palladium-catalyzed cross-coupling reactions

Abdelaziz Ejjoummany [1,2], Rabia Belaroussi [1,2], Ahmed El Hakmaoui [2], Mohamed Akssira [2,*], Gérald Guillaumet [1], Frédéric Buron [1] and Sylvain Routier [1,*]

1 Univ Orleans, CNRS, Institut de Chimie Organique et Analytique, UMR 7311, BP 6759, F-45067 Orléans Cedex 2, France. E-mail: abdelaziz.ejjoummany@univ-orleans.fr (A.E.); rabia.belaroussi@univ-orleans.fr (R.B.); gerald.guillaumet@univ-orleans.fr (G.G.); frederic.buron@univ-orleans.fr (F.B.); sylvain.routier@univ-orleans.fr (S.R.).

2 Laboratoire de Chimie Physique et Chimie Bioorganique, Université Hassan II- Casablanca, B. P. 146, 28800 Mohammedia, Morocco. E-mail: a_elhakmaoui@yahoo.fr (A.E-H.); mohamed.akssira@fstm.ac.ma (M.A.).

* Correspondence: mohamed.akssira@fstm.ac.ma; tel: +212-523-314-705. sylvain.routier@univ-orleans.fr.; Tel.: +33-238-494-853.

molecules

MDPI

Dimethyl Pyrazolo[1,5-a]pyridine-2,3 dicarboxylate **(2)**

^{1}HNMR (400 MHz, Chloroform-*d*).

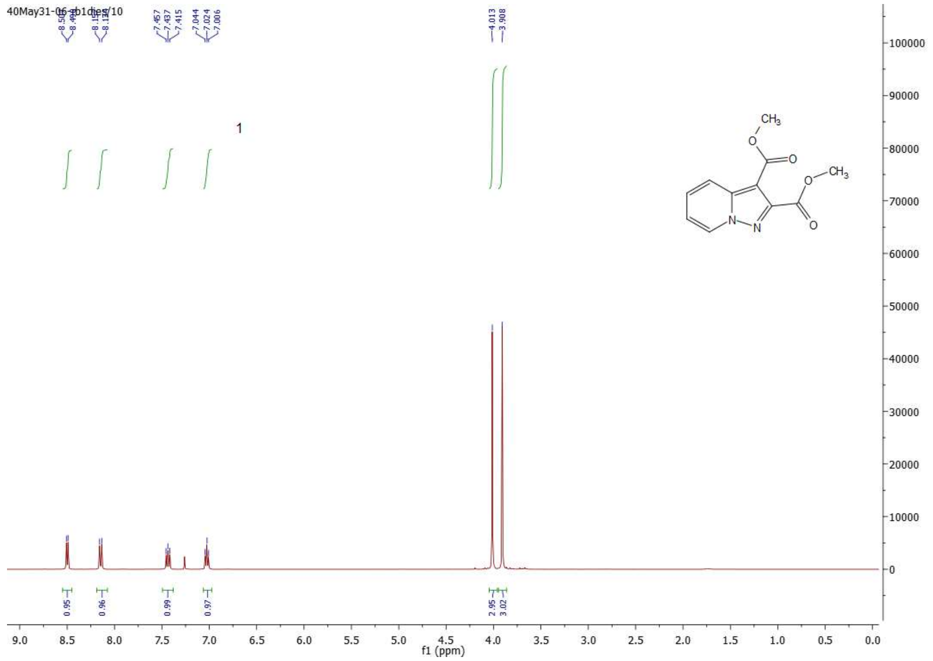

^{13}C NMR (101 MHz, $CDCl_3$).

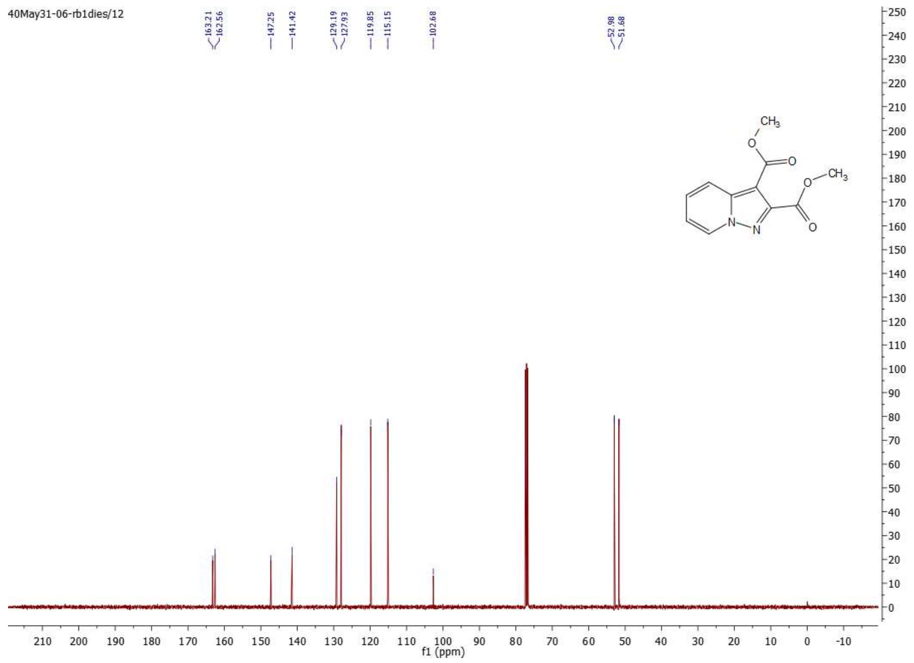

Pyrazolo [1, 5-a] pyridine-2-Dicarboxylic Acid **(3)**

^{1}H NMR (400 MHz, DMSO-d_6)

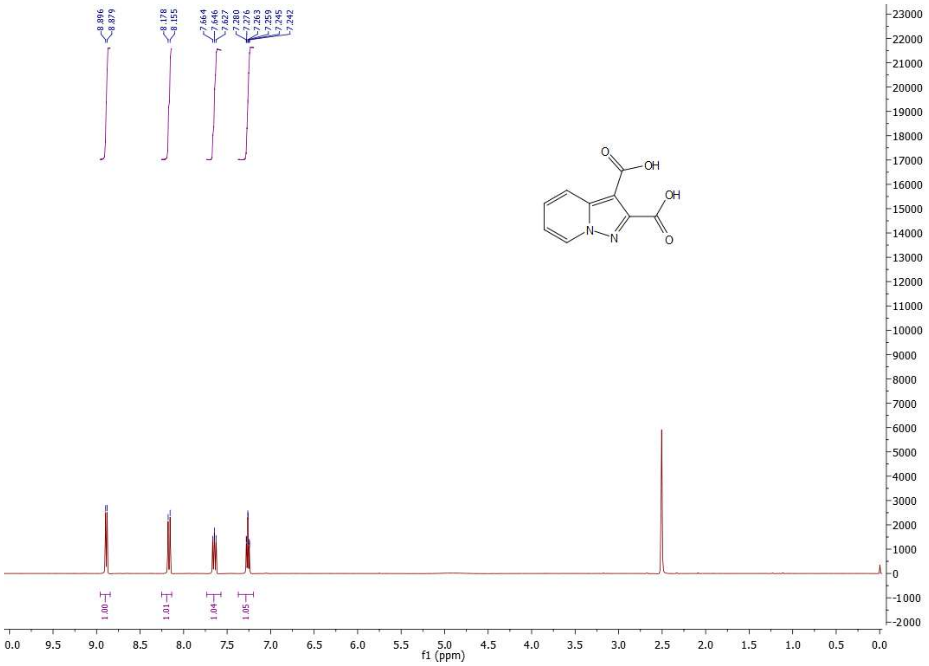

^{13}C NMR (101 MHz, DMSO-d_6)

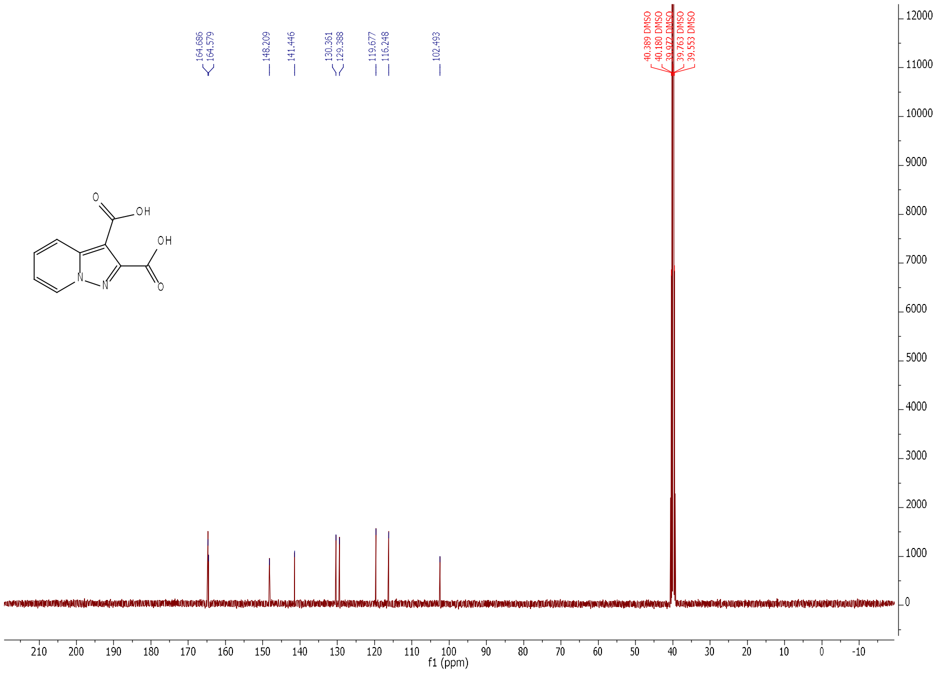

molecules

2-(Methoxycarbonyl)pyrazolo[1,5-a]pyridine-3-carboxylic Acid **(4)**

^{1}H NMR (400 MHz, DMSO-d_6)

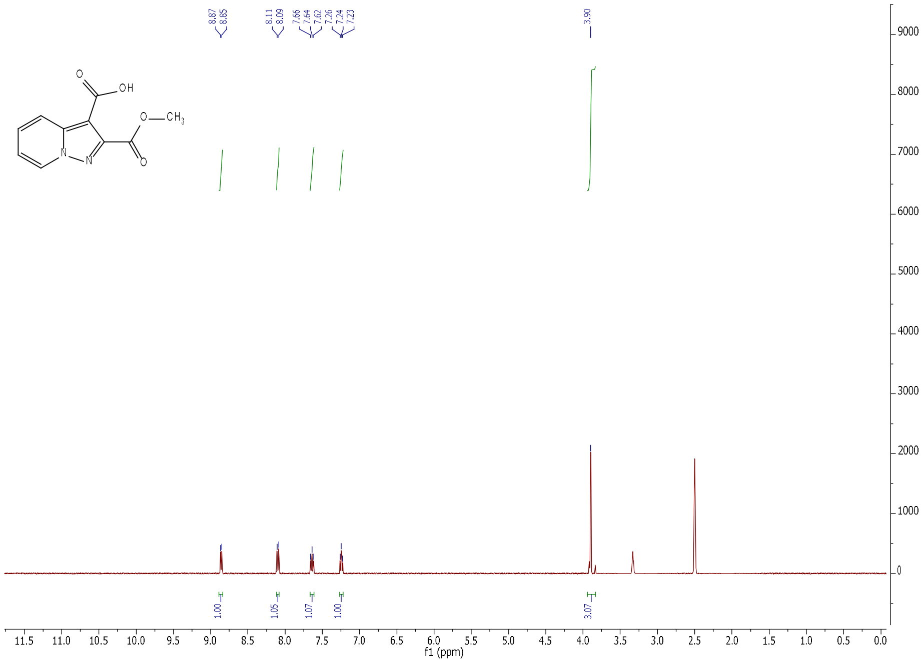

^{13}C NMR (101 MHz, DMSO-d_6)

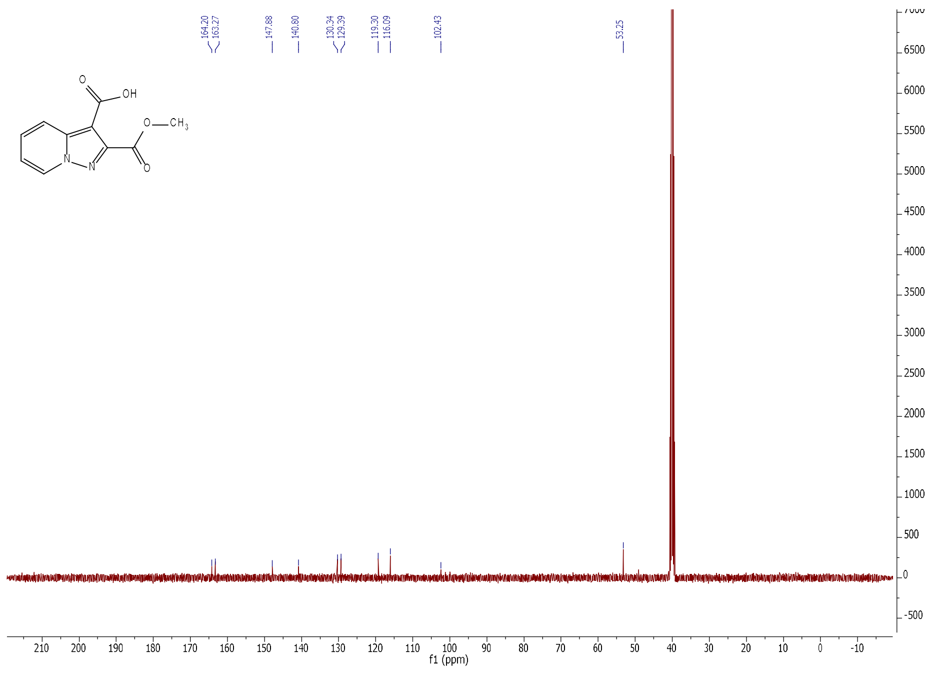

molecules

MDPI

Methyl 3-(tert-Butoxycarbonylamino)pyrazolo[1,5-a]pyridine- 2-carboxylate **(5)**

^{1}H NMR (400 MHz, Chloroform-*d*)

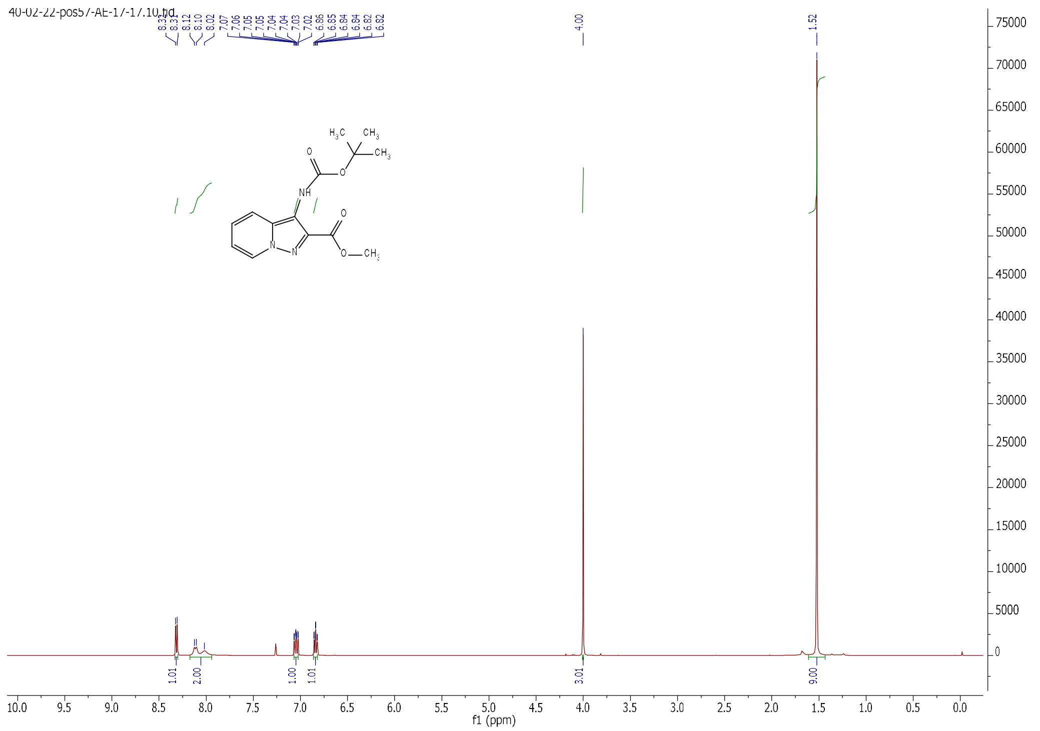

^{13}C NMR (101 MHz, $CDCl_3$).

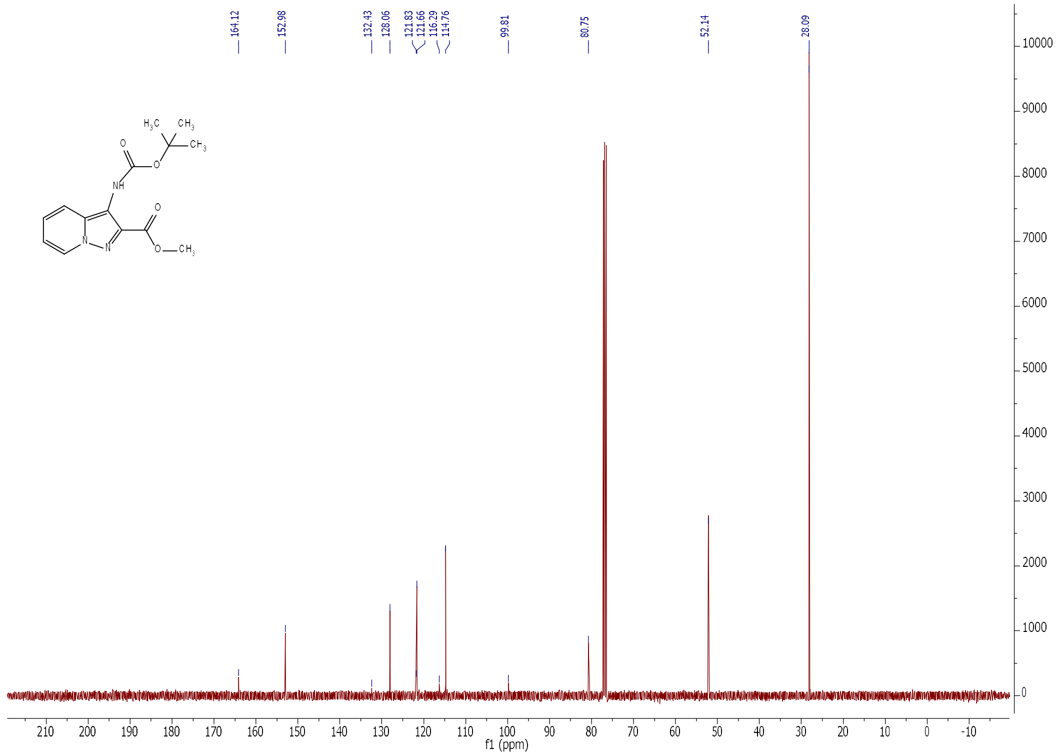

Methyl 3-aminopyrazolo [1, 5-a] pyridine-2-carboxylate **(6)**

^{1}H NMR (400 MHz, Chloroform-*d*)

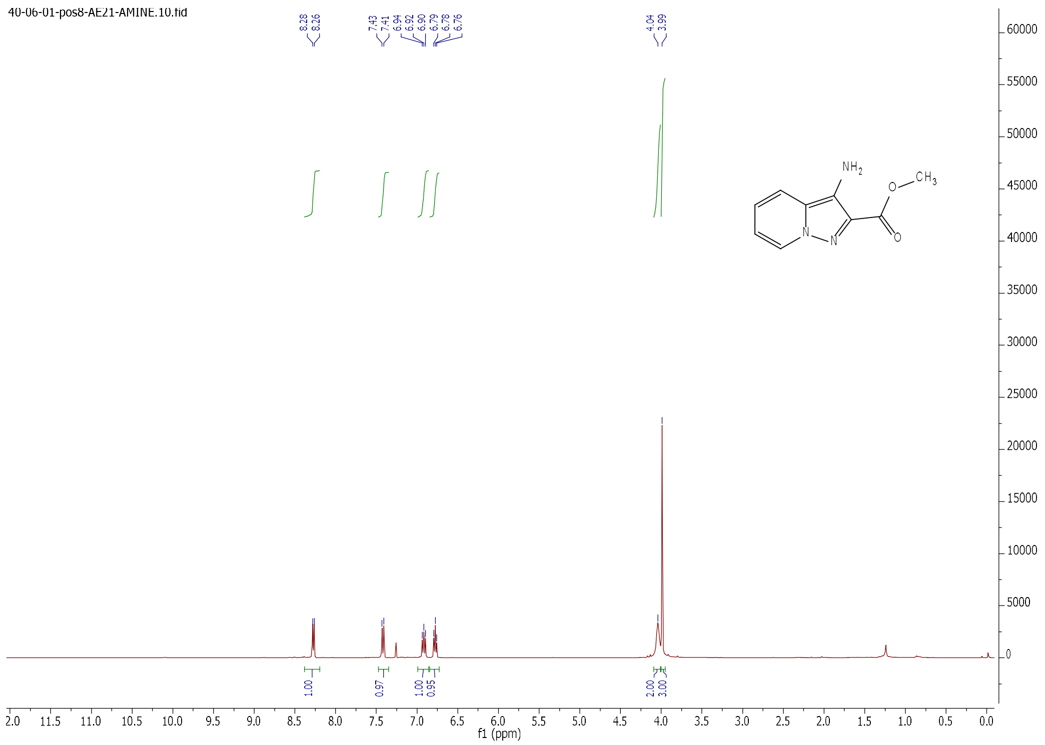

^{13}C NMR (101 MHz, $CDCl_3$).

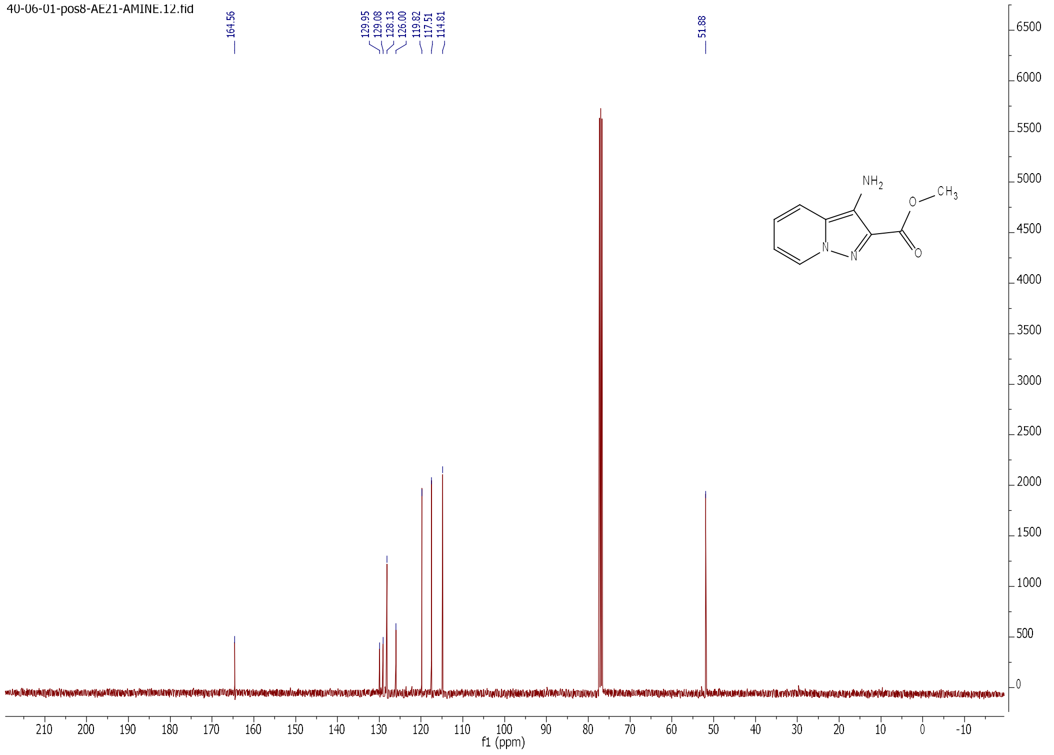

molecules

MDPI

Methyl 3-[(3-oxo-3-phenyl-propanethioyl) amino] pyrazolo [1, 5-a] pyridine-2-carboxylate **(7)**

^{1}H NMR (250 MHz, DMSO-*d6*)

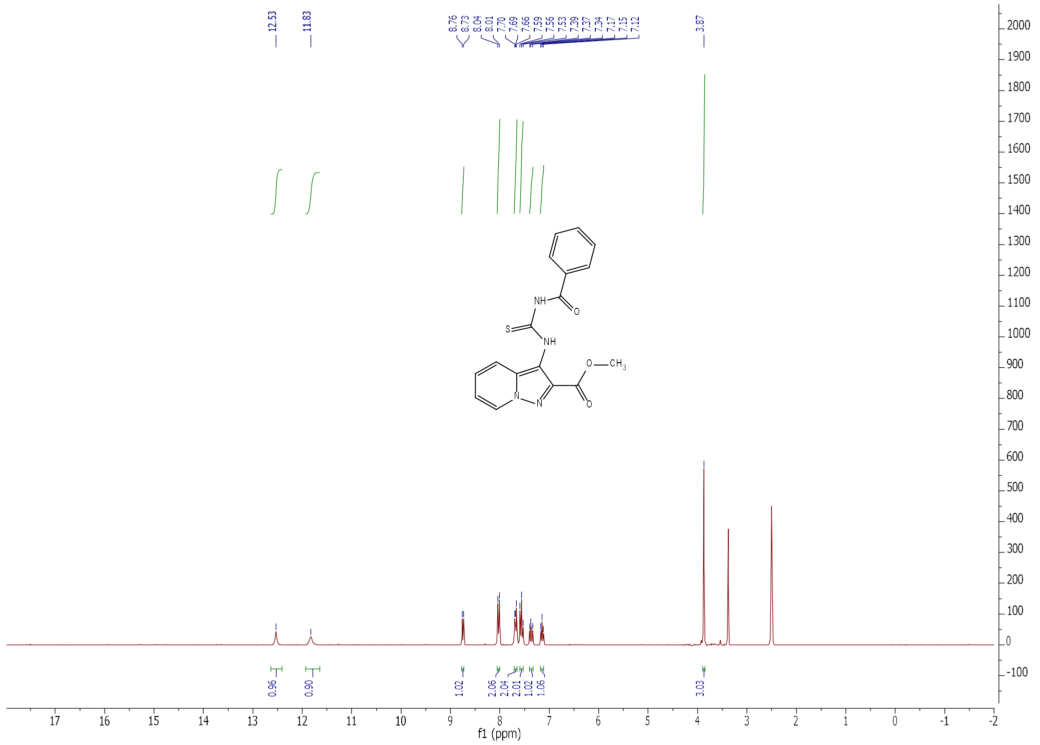

^{13}C NMR (101 MHz, DMSO-d_6)

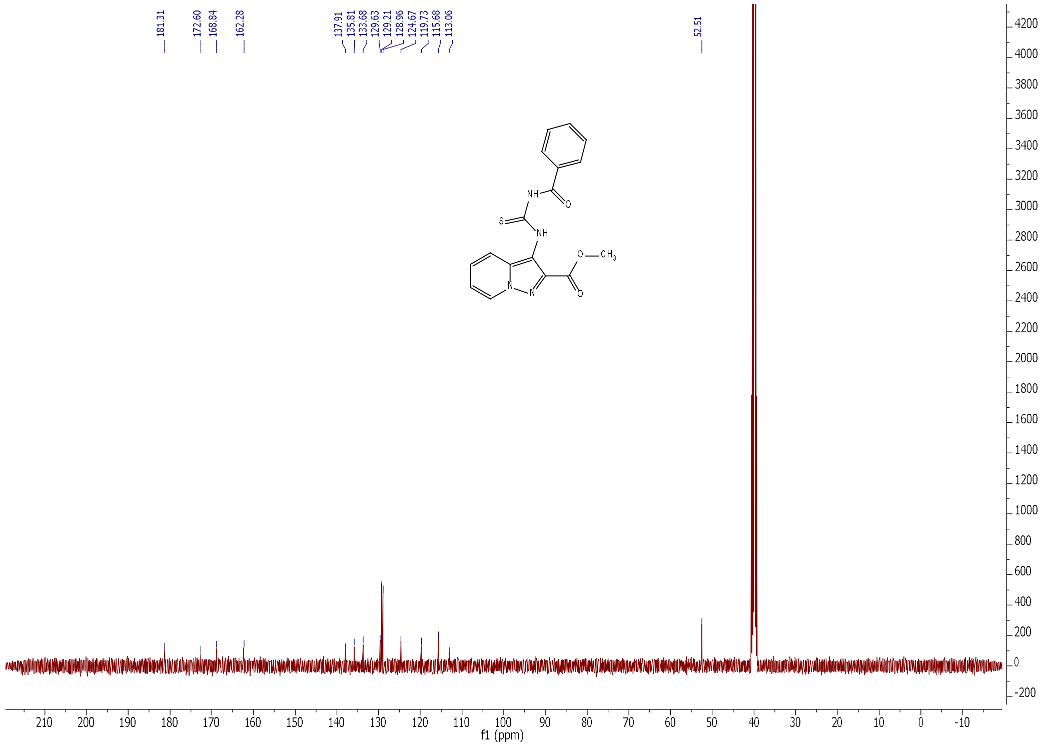

molecules

MDPI

2-Thioxo-1H-pyrido [3, 4]pyrazolo[1,3-b]pyrimidin-4-one **(8)**

^{1}H NMR (250 MHz, DMSO-d_6)

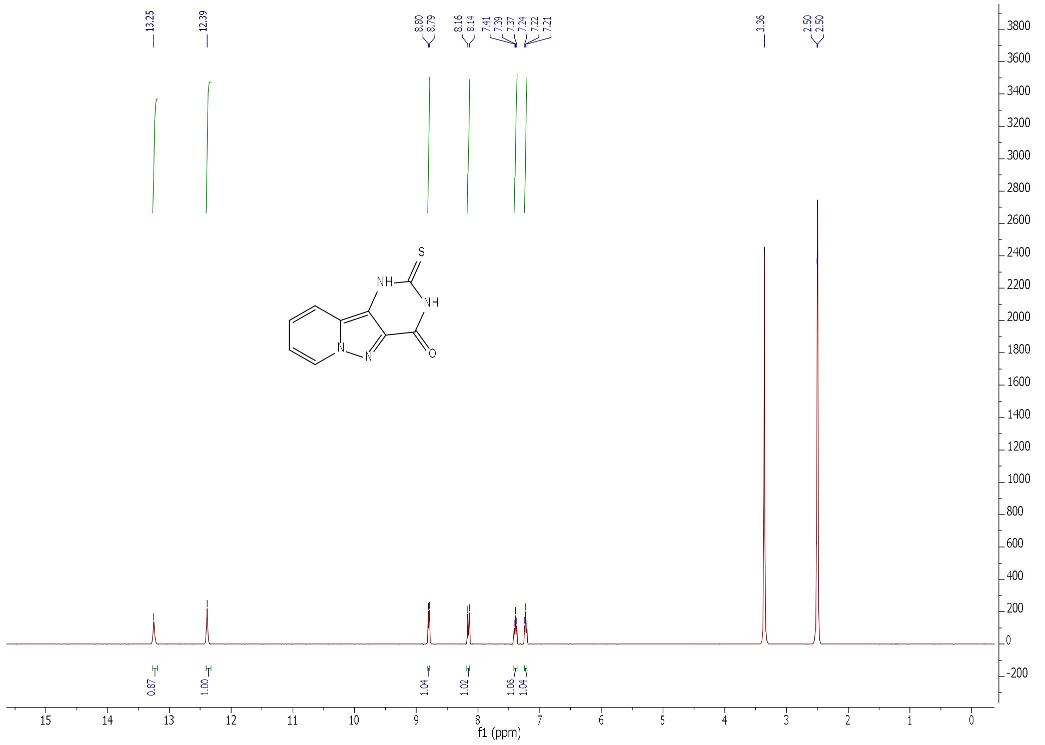

^{13}C NMR (101 MHz, DMSO-d_6)

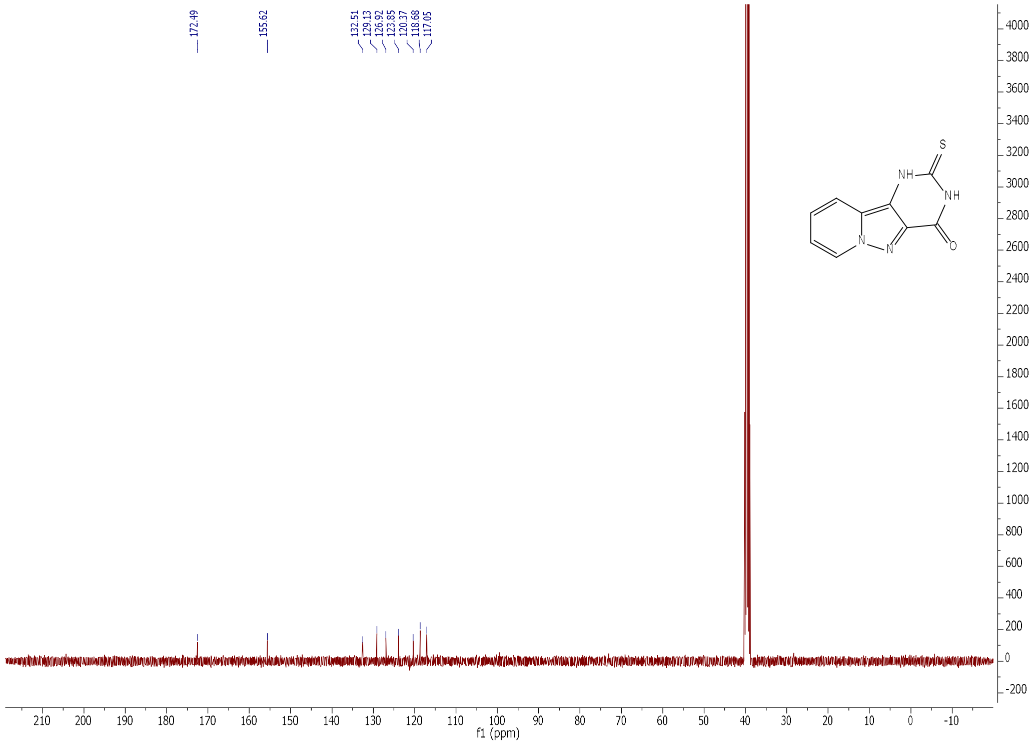

molecules

MDPI

2-methylsulfanyl-3H-pyrido[3,4]pyrazolo[1,3-b]pyrimidin-4-one (**9**)

^{1}H NMR (250 MHz, DMSO-d_6)

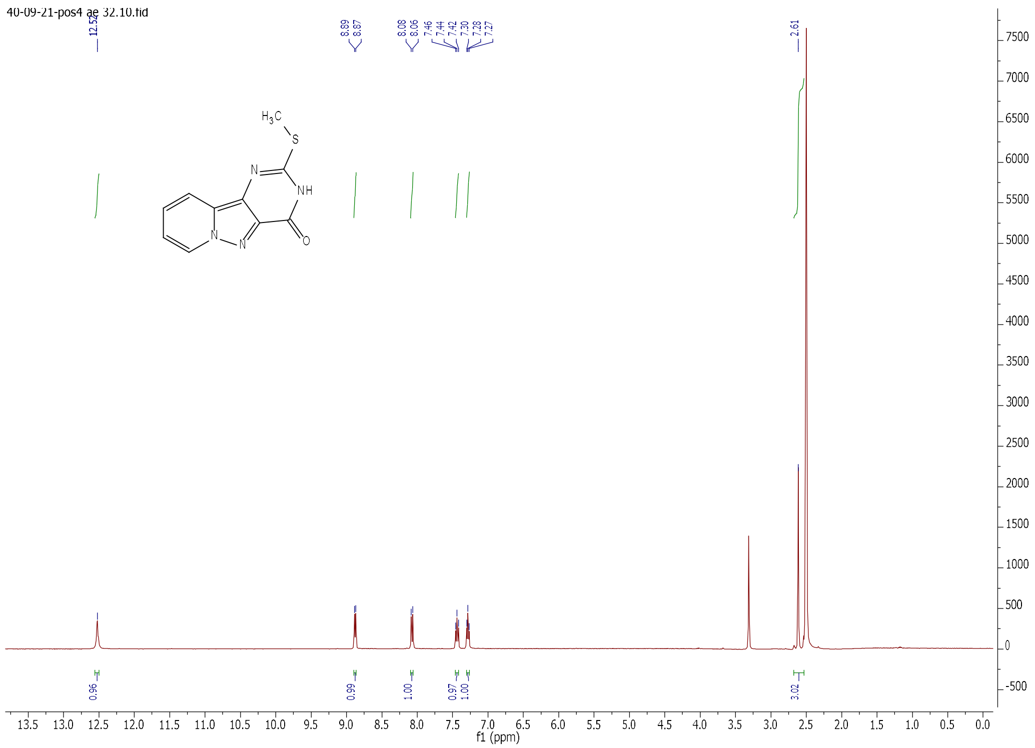

^{13}C NMR (101 MHz, DMSO-d_6)

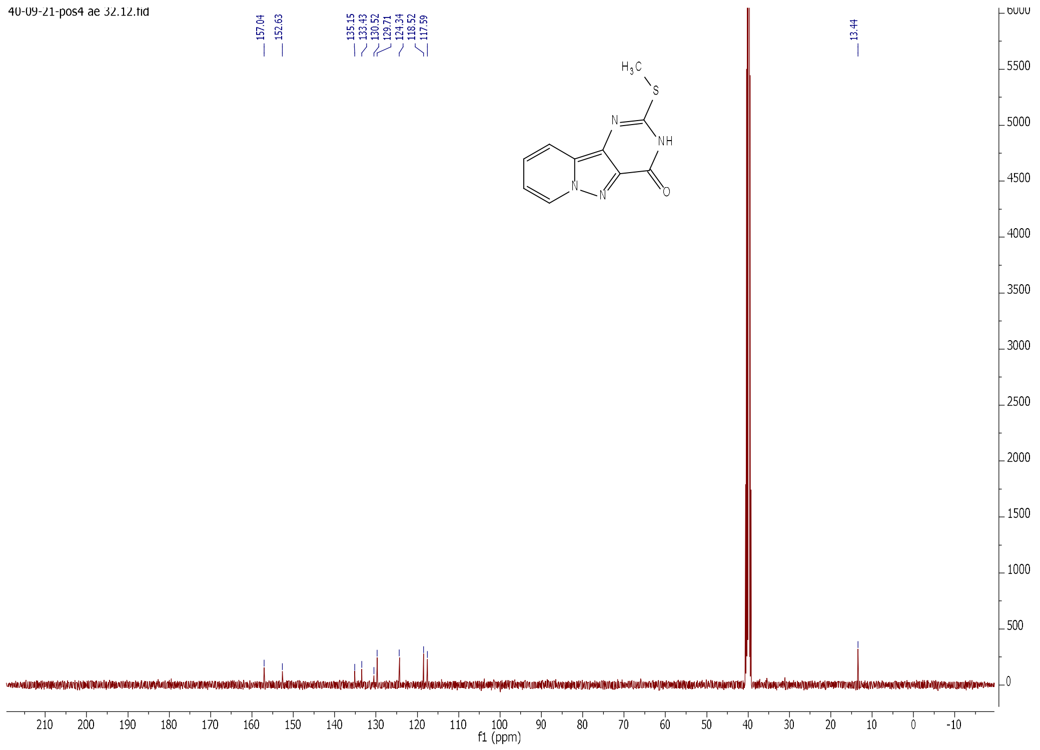

molecules

MDPI

2-methylsulfanyl-4-(p-tolyl)pyrido[3,4]pyrazolo[1,3-b]pyrimidine **(10)**

^{1}H NMR (400 MHz, Chloroform-*d*)

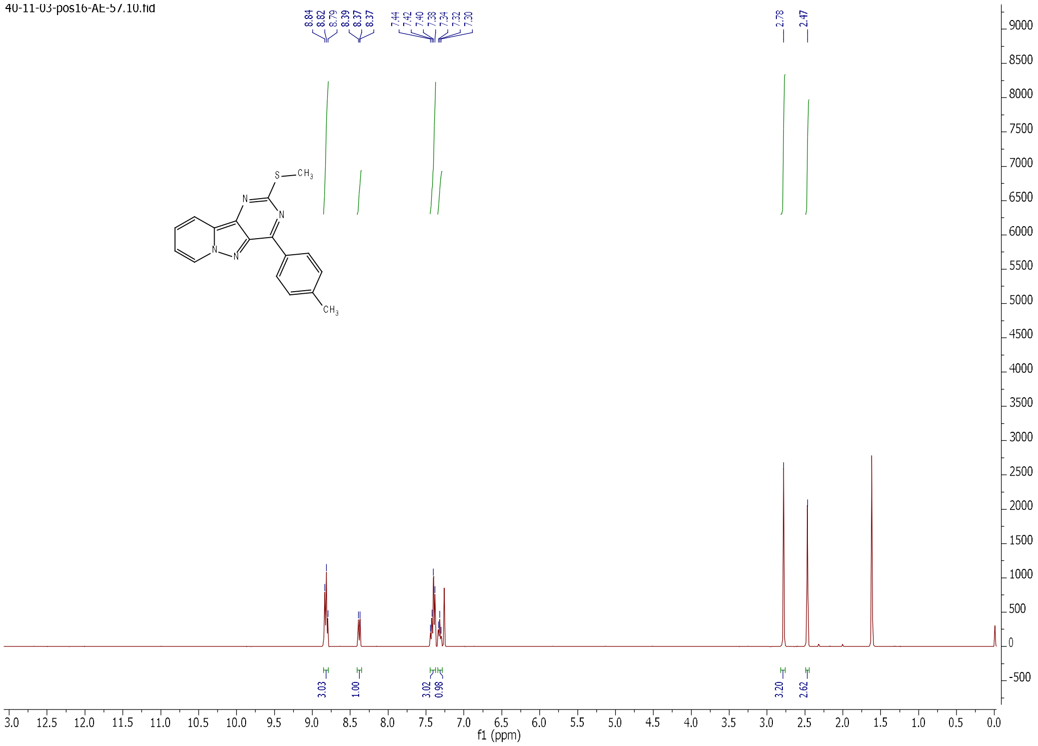

^{13}C NMR (101 MHz, $CDCl_3$)

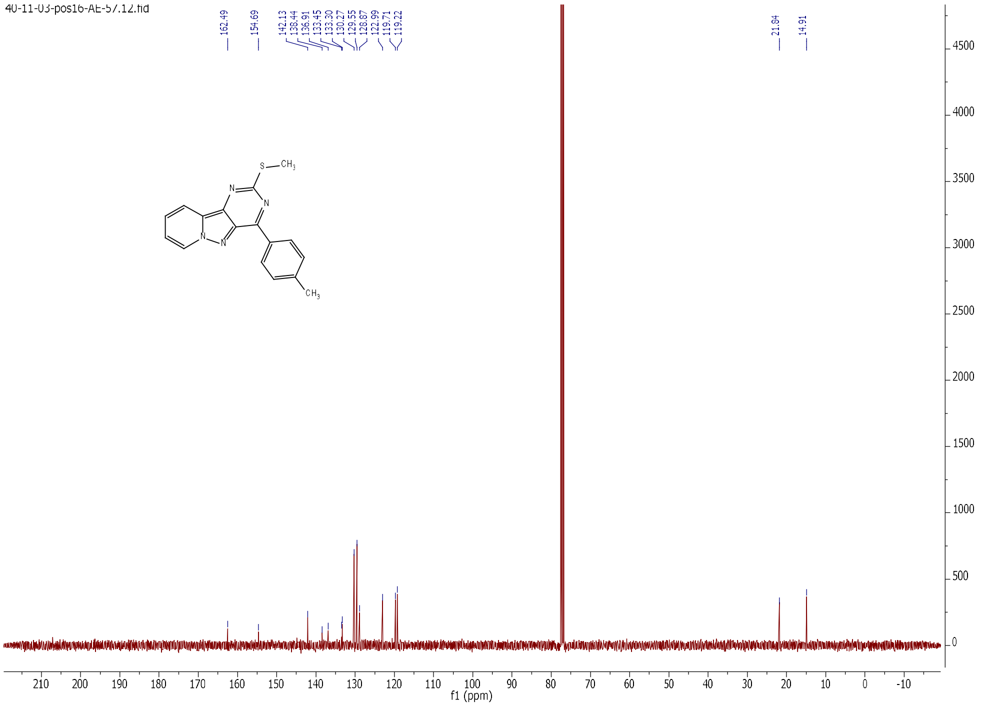

4-(4-methoxyphenyl)-2-methylsulfanyl-pyrido[3,4]pyrazolo[1,3-b]pyrimidine **(11)**

^{1}H NMR (250 MHz, $CDCl_3$)

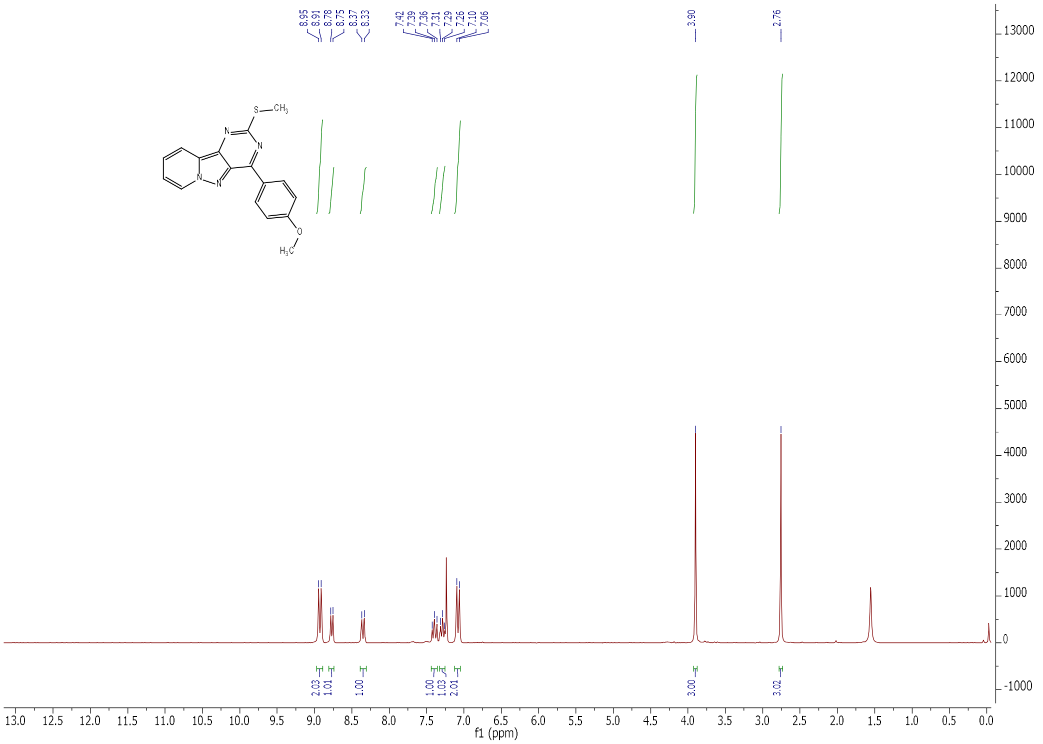

^{13}C NMR (101 MHz, $CDCl_3$)

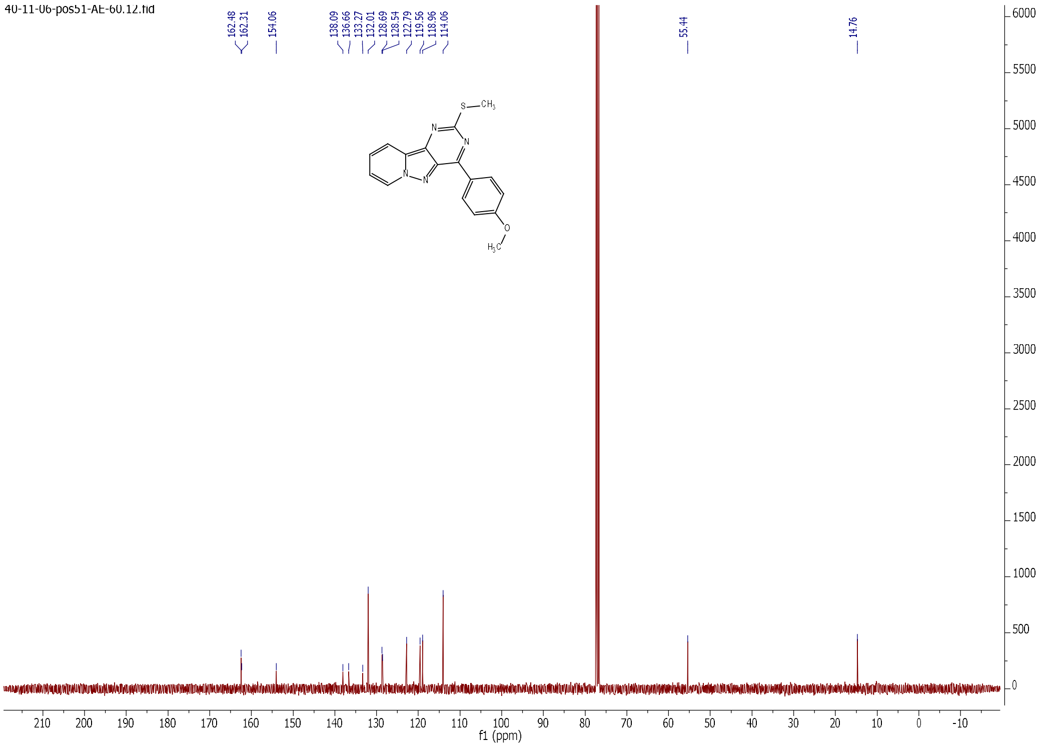

molecules

4-(3-methoxyphenyl)-2-methylsulfanyl-pyrido[3,4]pyrazolo[1,3-b]pyrimidine **(12)**

^{1}H NMR (250 MHz, Chloroform-*d*)

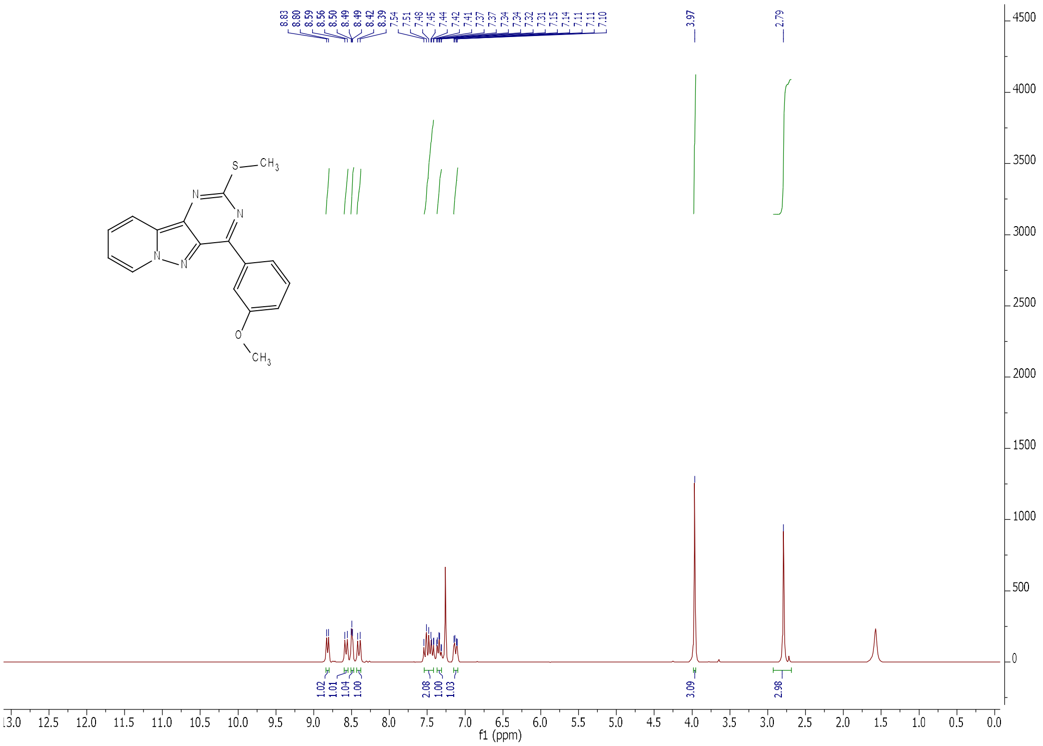

^{13}C NMR (101 MHz, $CDCl_3$)

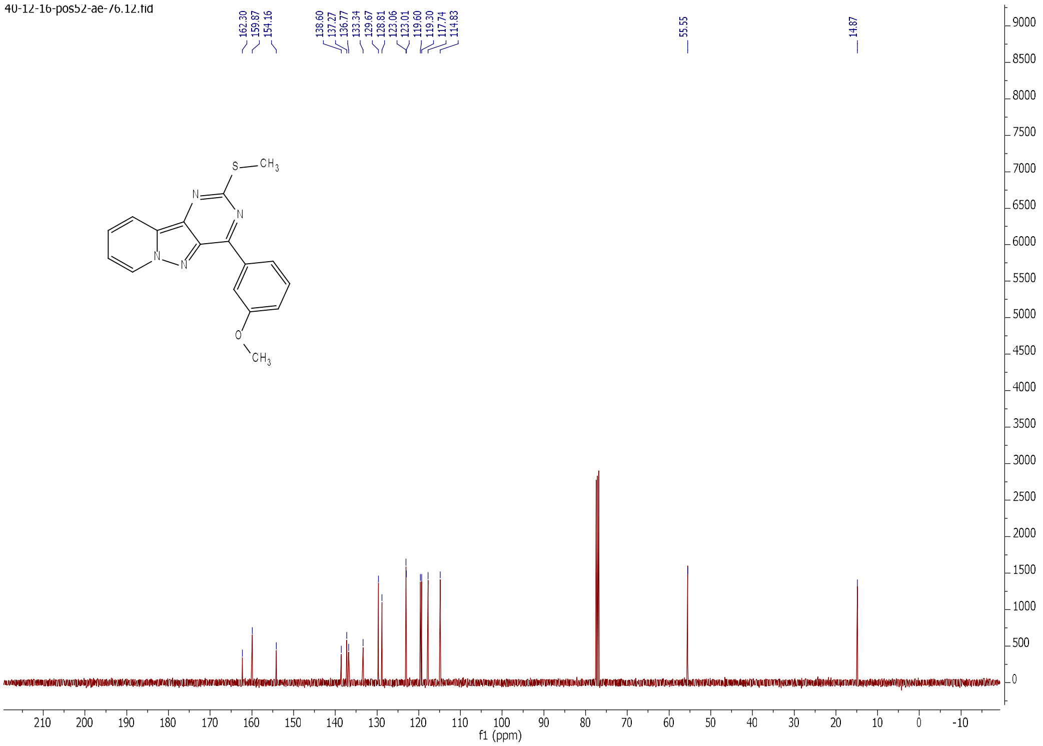

molecules

MDPI

4-(2-methoxyphenyl)-2-methylsulfanyl-pyrido[3,4]pyrazolo[1,3-b]pyrimidine **(13)**

^{1}H NMR (250 MHz, Chloroform-*d*)

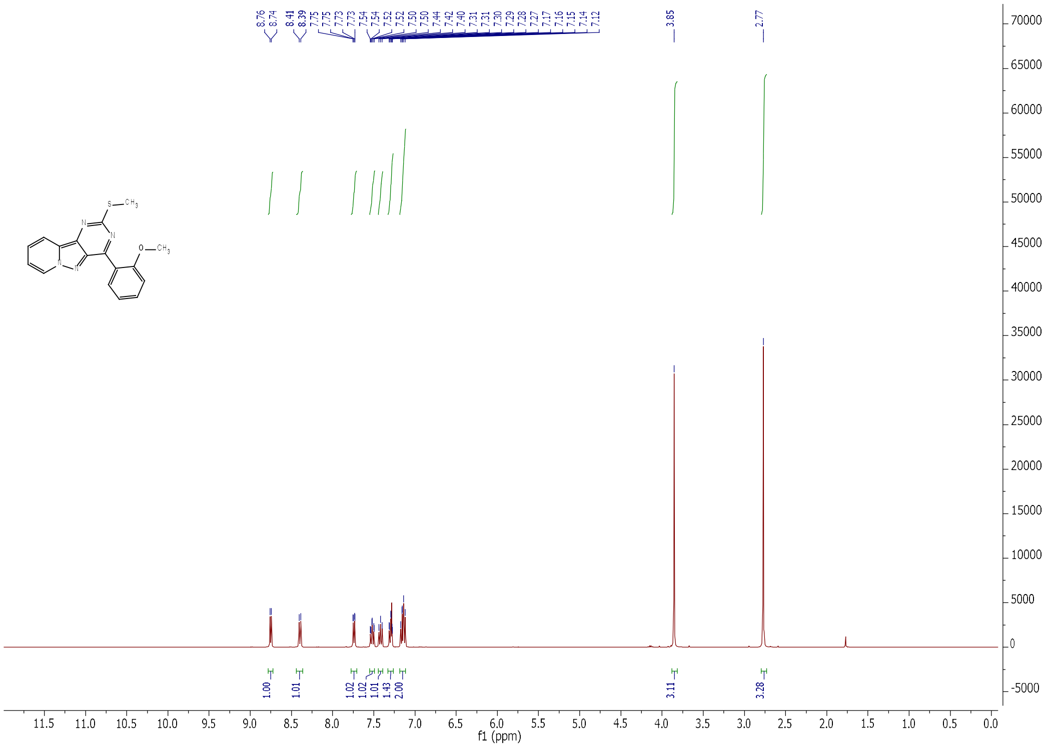

^{13}C NMR (101 MHz, $CDCl_3$)

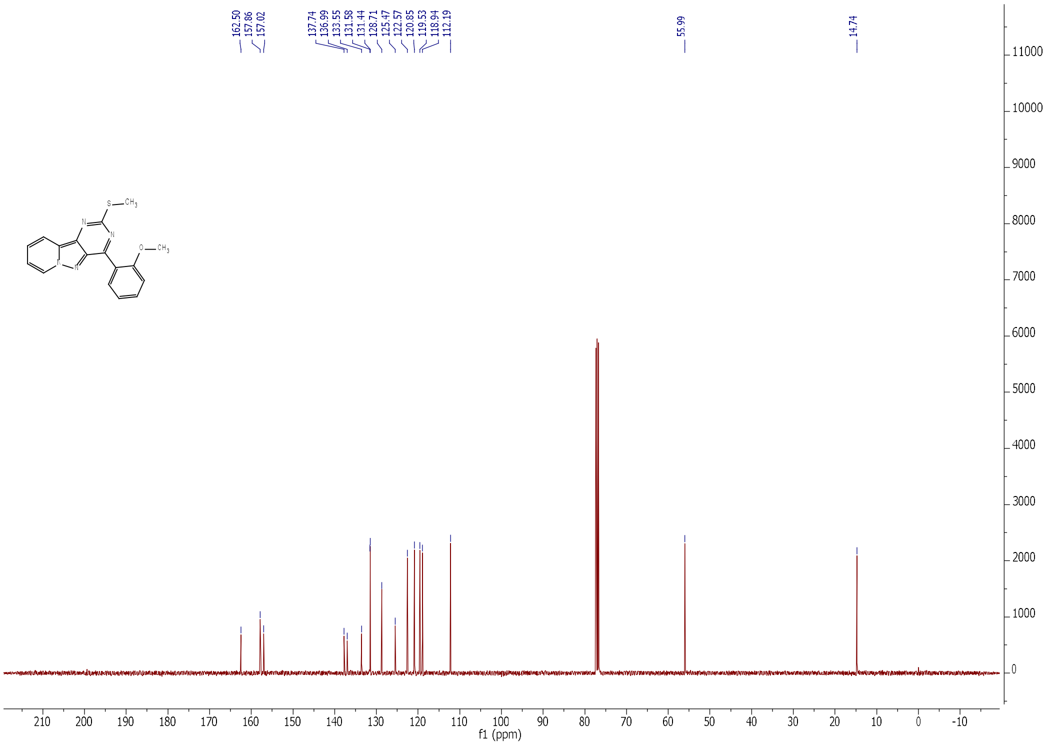

molecules

MDPI

4-(4-fluorophenyl)-2-methylsulfanyl-pyrido[3,4]pyrazolo[1,3-b]pyrimidine **(14)**

^{1}H NMR (250 MHz, Chloroform-*d*)

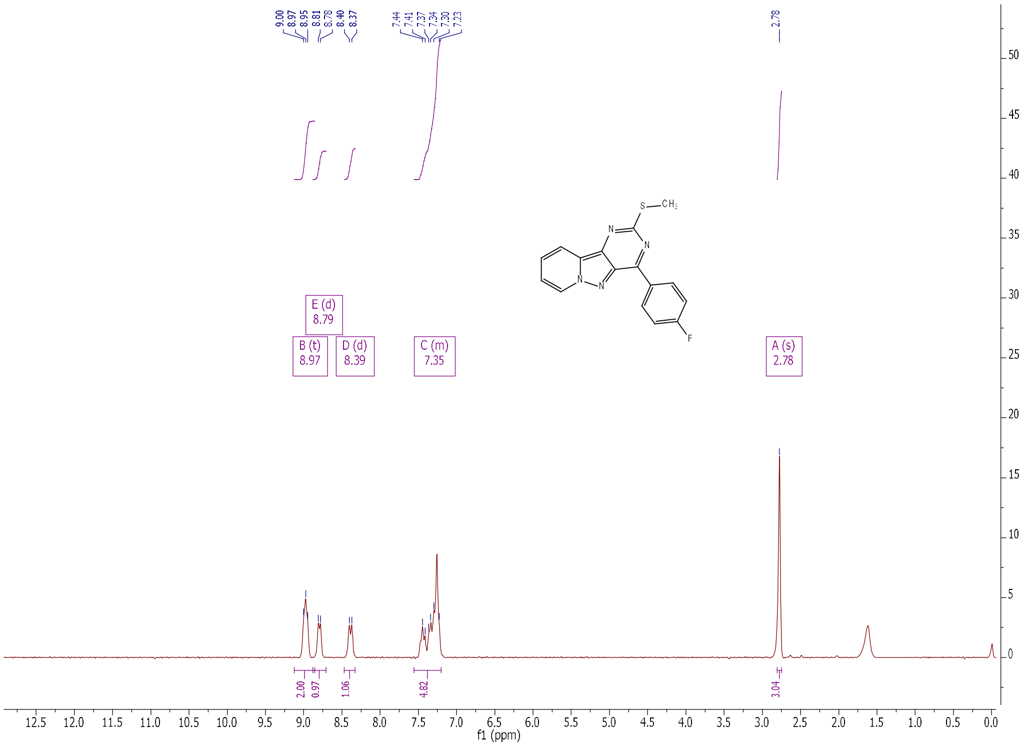

^{13}C NMR (101 MHz, $CDCl_3$)

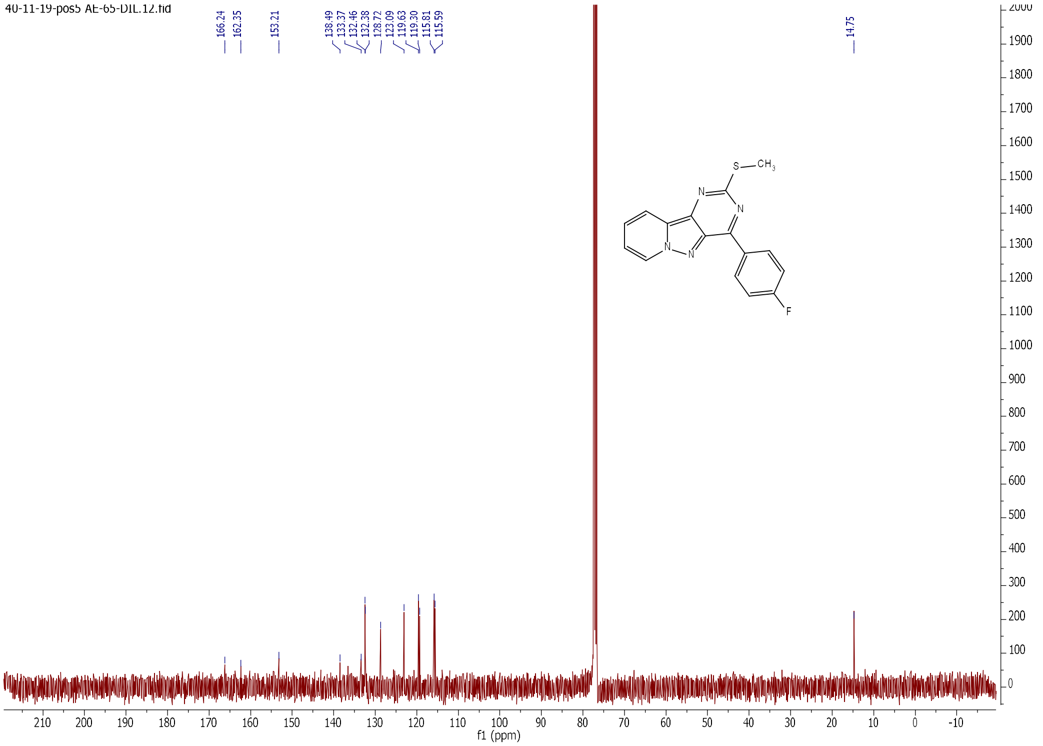

molecules

MDPI

2-methylsulfanyl-4-[4-(trifluoromethyl)phenyl]pyrido[3,4]pyrazolo[1,3-b]pyrimidine **(15)**

^{1}H NMR (400 MHz, Chloroform-*d*)

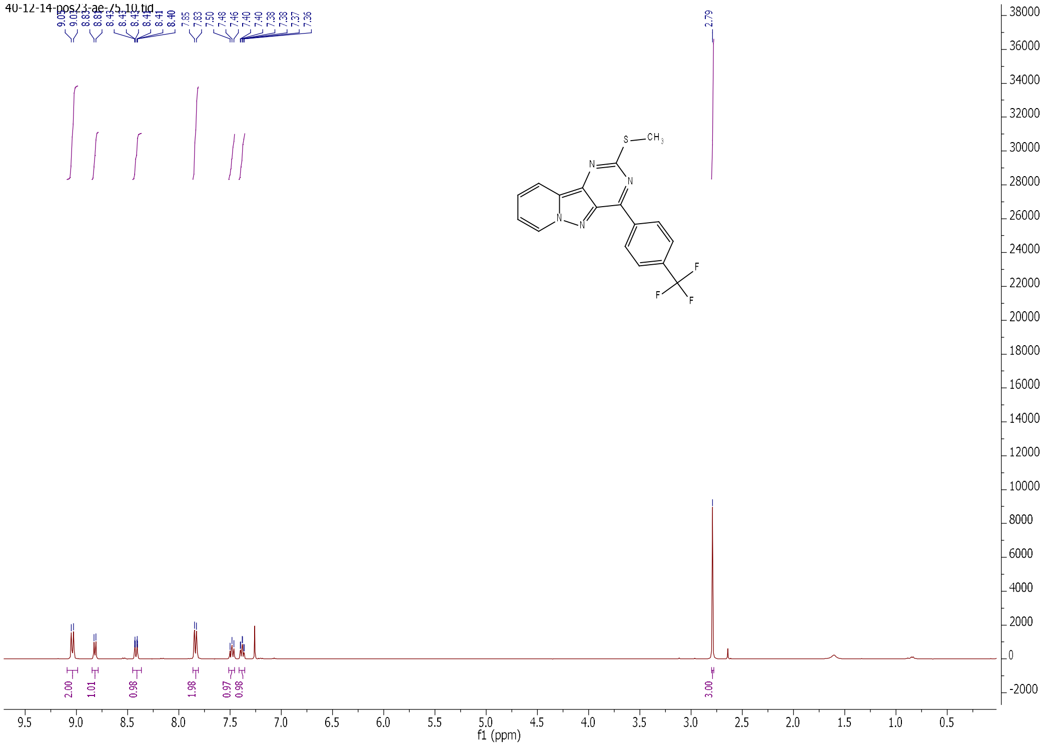

^{13}C NMR (101 MHz, $CDCl_3$)

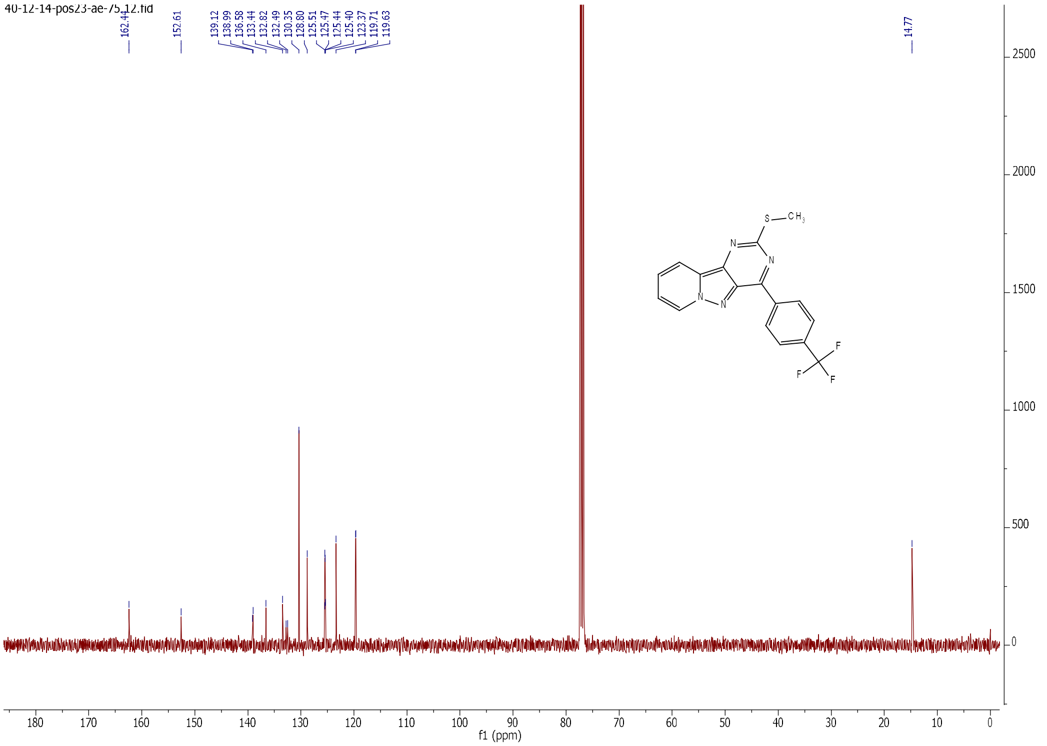

molecules

MDPI

4-(2-methylsulfanylpyrido[3,4]pyrazolo[1,3-b]pyrimidin-4-yl)benzonitrile **(16)**

^{1}H NMR (400 MHz, Chloroform-*d*)

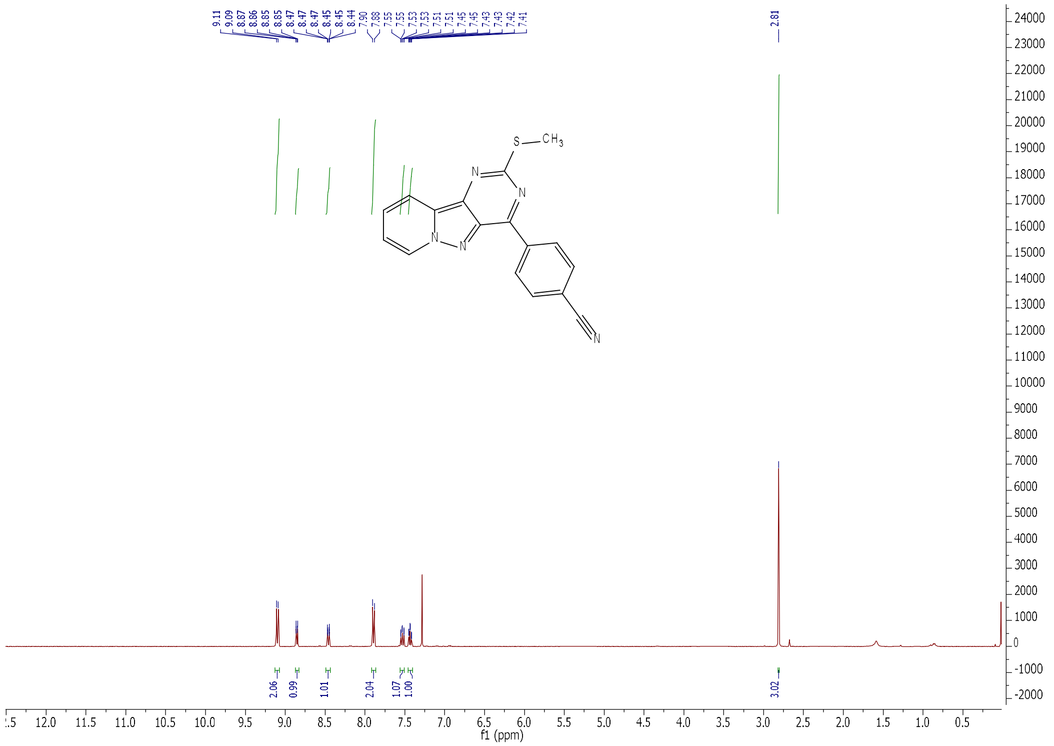

^{13}C NMR (101 MHz, $CDCl_3$)

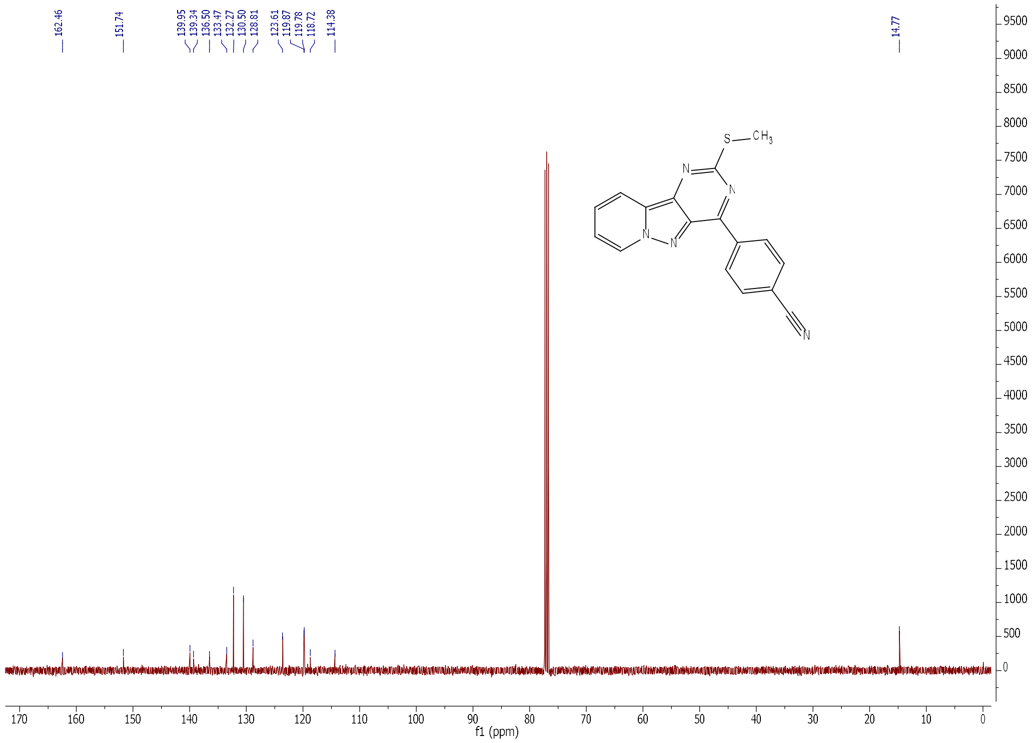

molecules

MDPI

4-(2-methylsulfanylpyrido[3,4]pyrazolo[1,3-b]pyrimidin-4-yl)phenol **(17)**

^{1}H NMR (250 MHz, Chloroform-*d*)

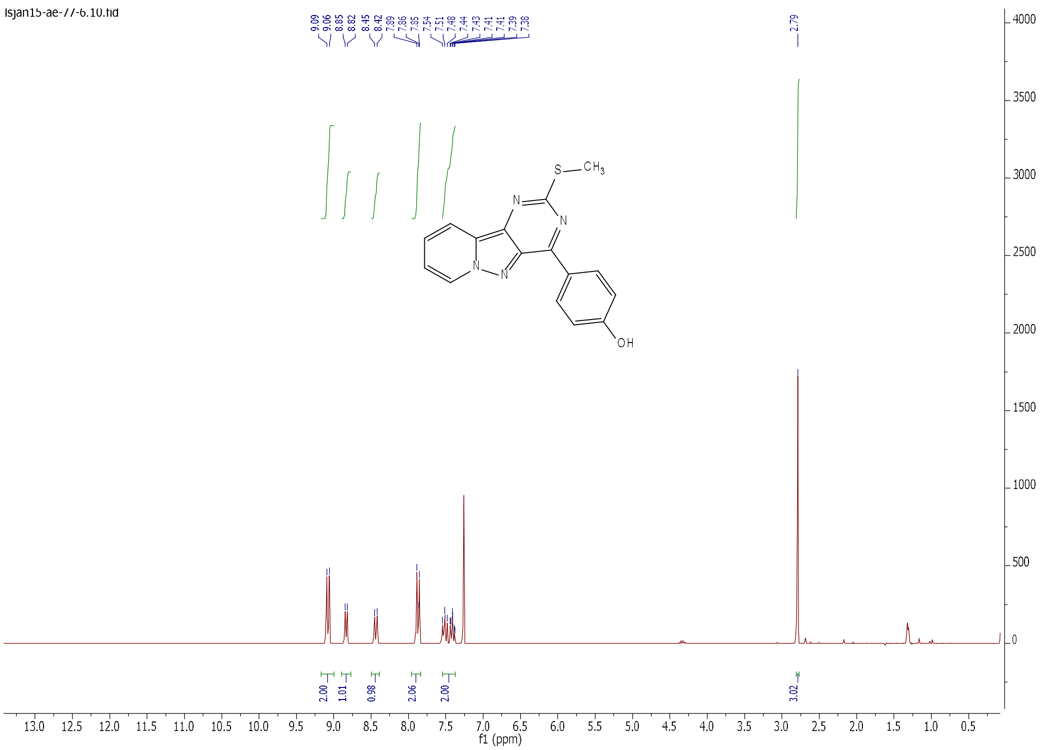

^{13}C NMR (101 MHz, $CDCl_3$)

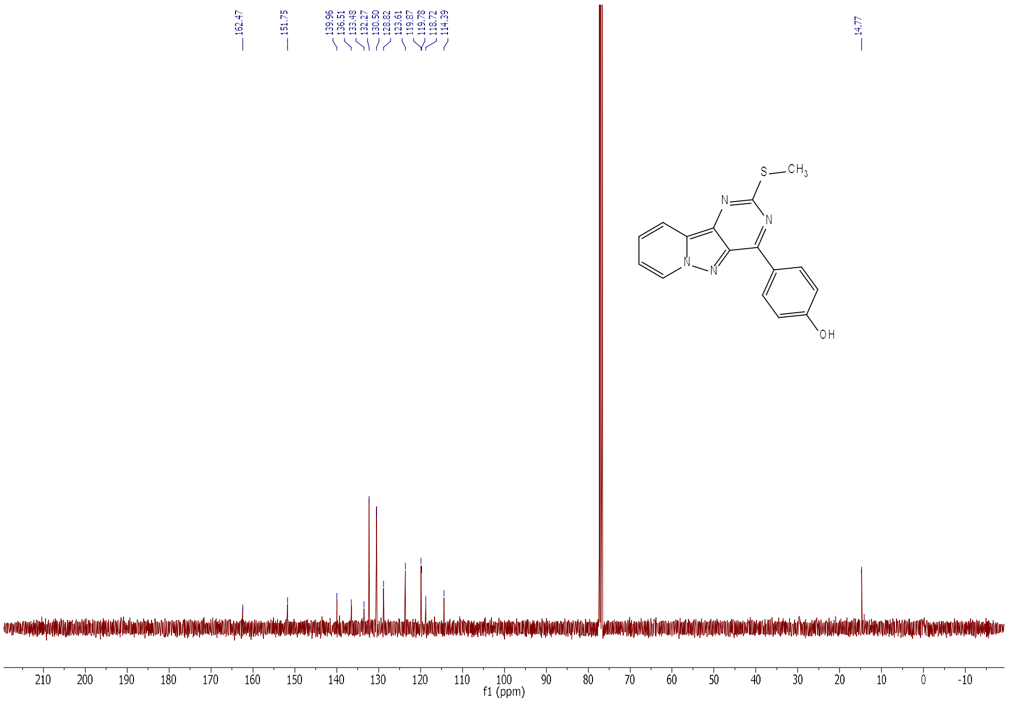

molecules

MDPI

4-(2-furyl)-2-methylsulfanyl-pyrido[3,4]pyrazolo[1,3-b]pyrimidine **(18)**

^{1}H NMR (400 MHz, Chloroform-*d*)

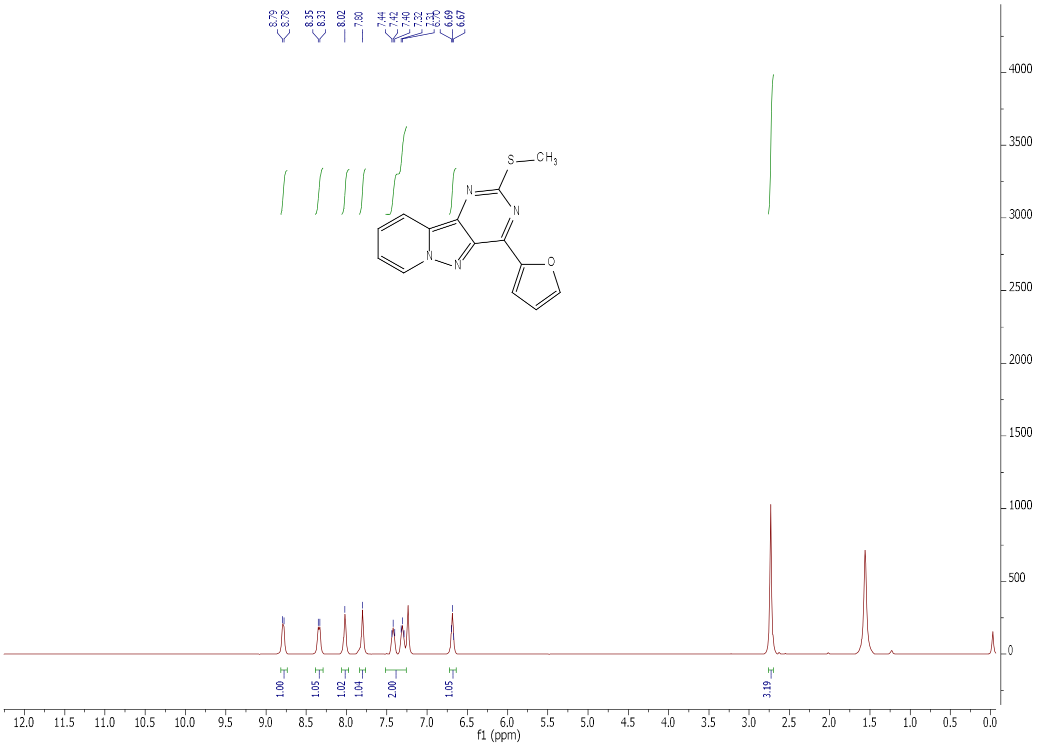

^{13}C NMR (101 MHz, $CDCl_3$)

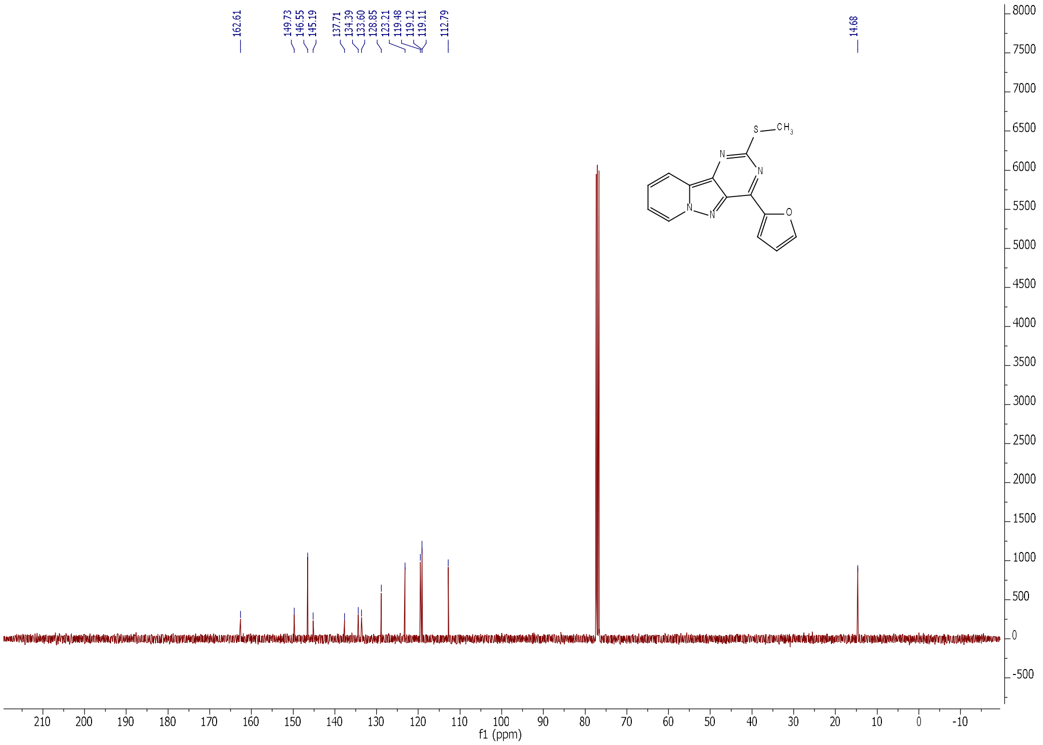

2,4-bis(p-tolyl)pyrido[3,4]pyrazolo[1,3-b]pyrimidine **(20)**

^{1}H NMR 400 MHz, Chloroform-*d*)

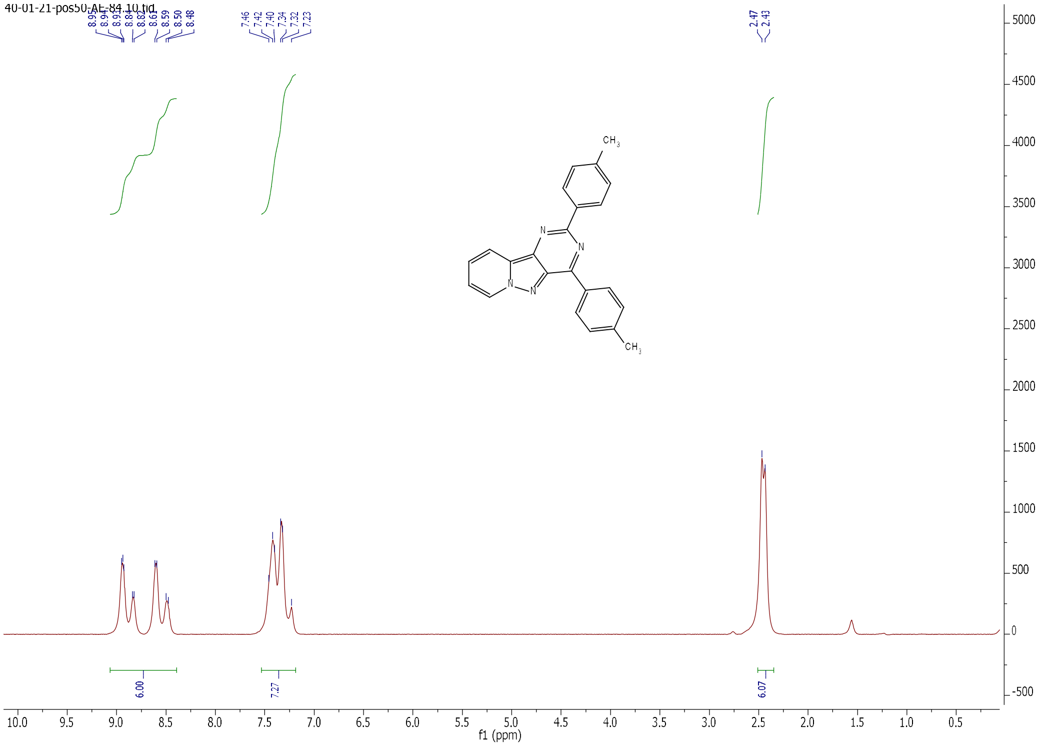

^{13}C NMR (101 MHz, $CDCl_3$)

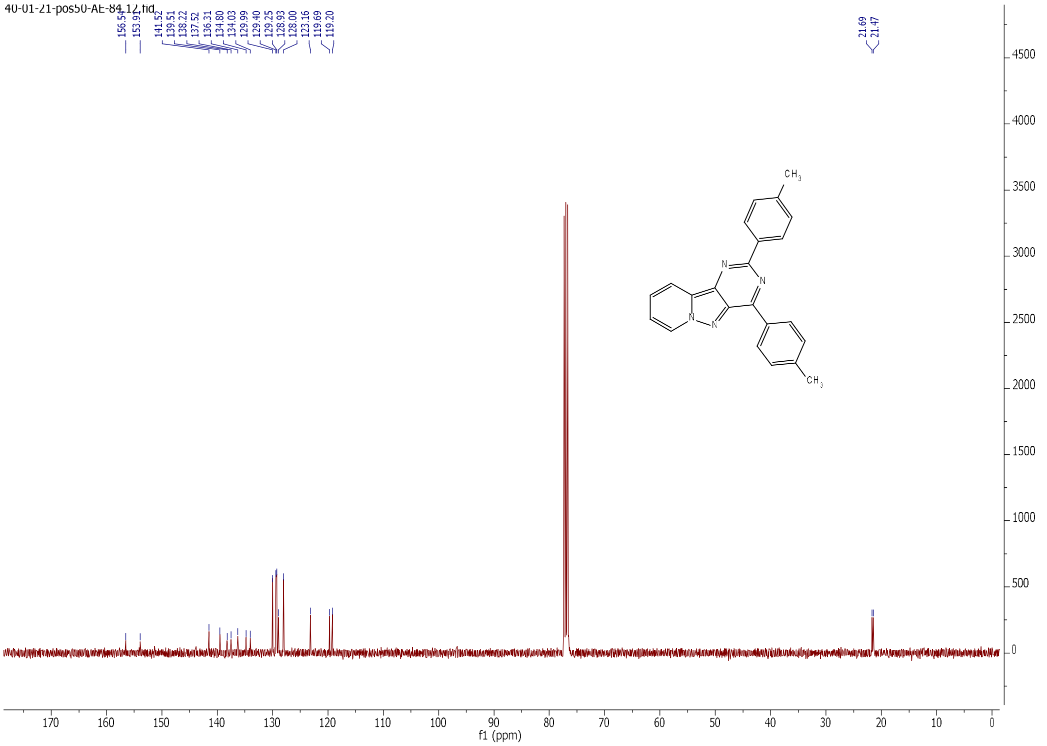

molecules

MDPI

2-(4-methoxyphenyl)-4-(p-tolyl)pyrido[3,4]pyrazolo[1,3-b]pyrimidine **(21)**

^{1}H NMR (400 MHz, Chloroform-*d*)

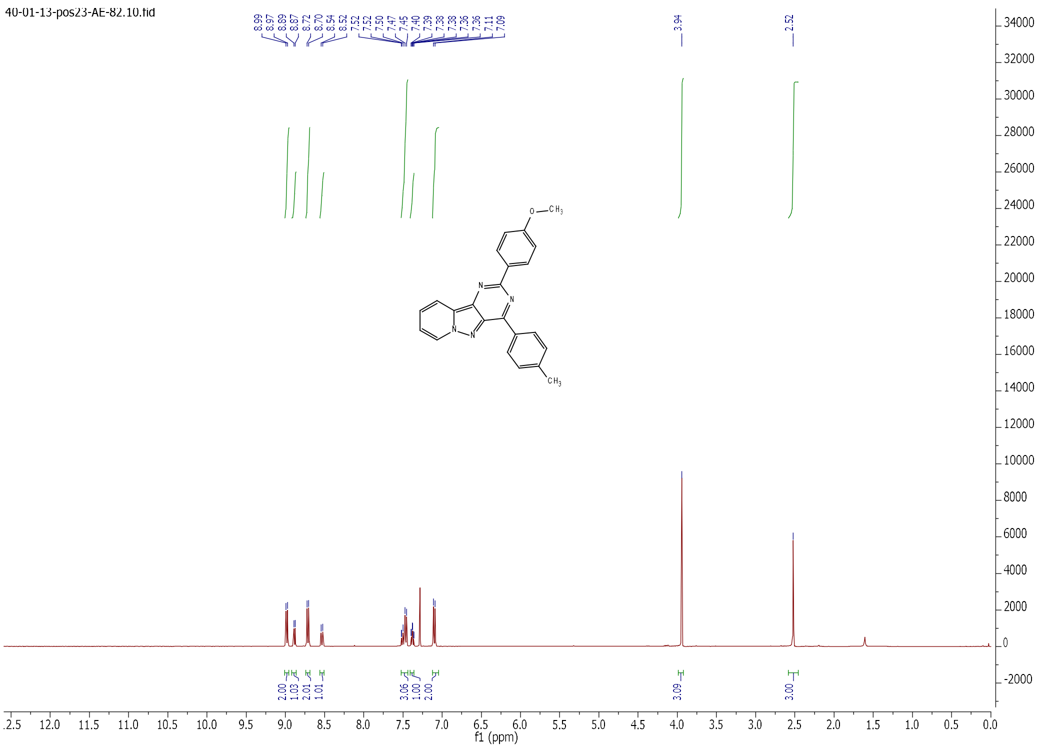

^{13}C NMR (101 MHz, $CDCl_3$)

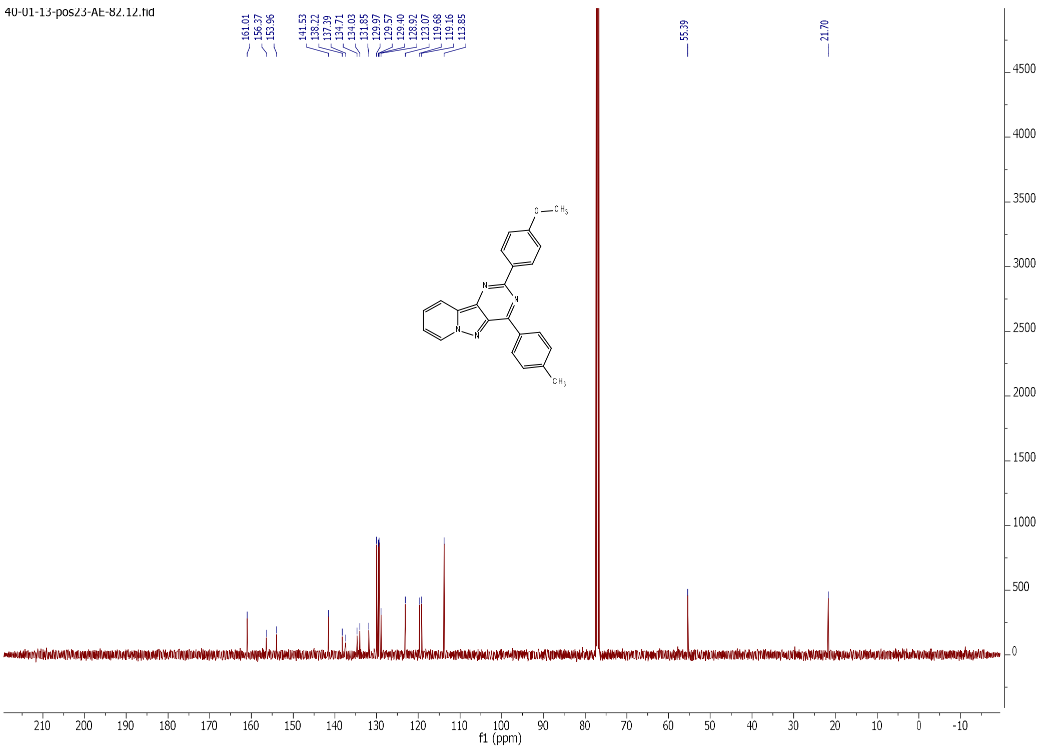

molecules

MDPI

2-(3-methoxyphenyl)-4-(p-tolyl)pyrido[3,4]pyrazolo[1,3-b]pyrimidine **(22)**

^{1}H NMR (250 MHz, Chloroform-*d*)

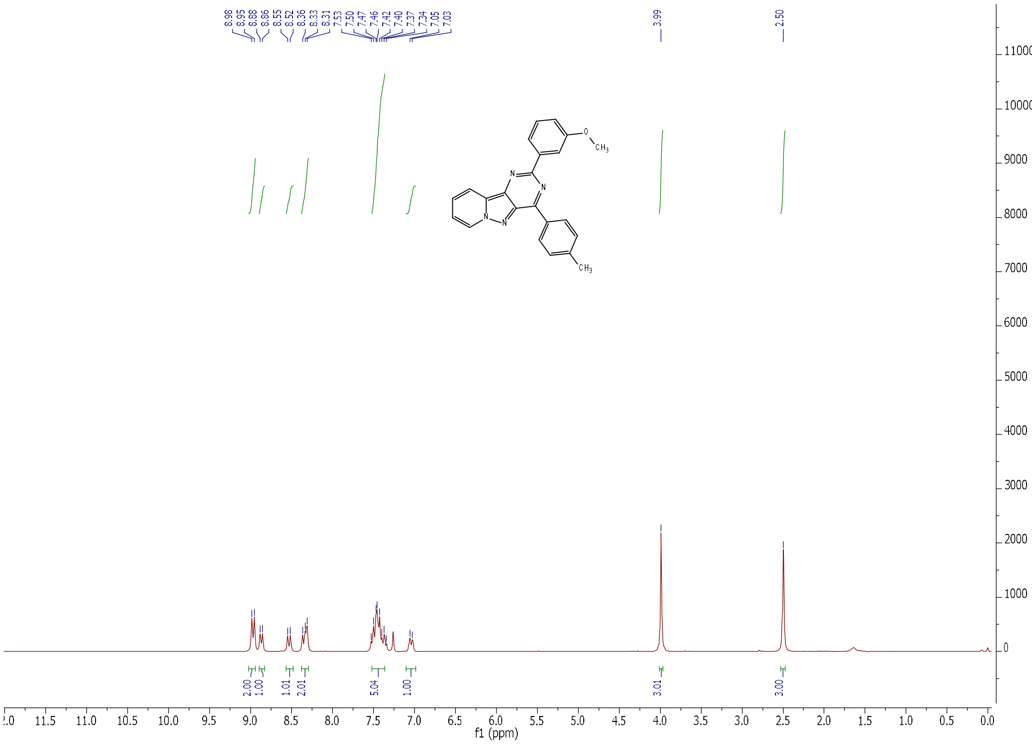

^{13}C NMR (101 MHz, $CDCl_3$)

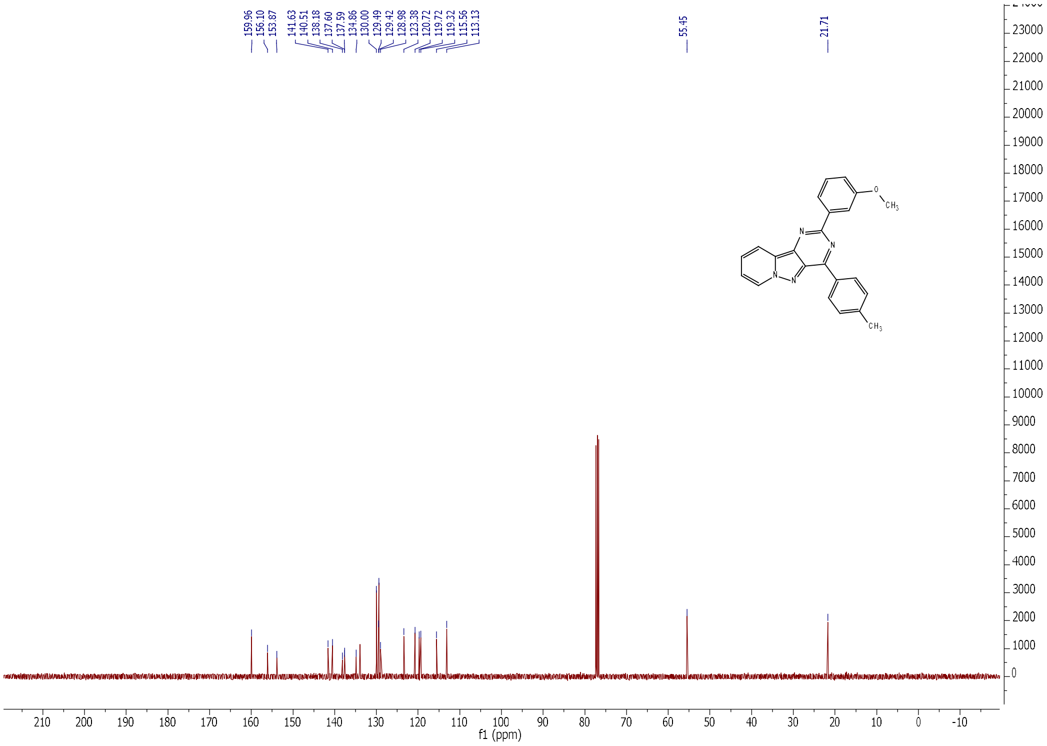

molecules
MDPI

2-(2-methoxyphenyl)-4-(p-tolyl)pyrido[3,4]pyrazolo[1,3-b]pyrimidine **(23)**

^{1}H NMR (250 MHz, Chloroform-*d*)

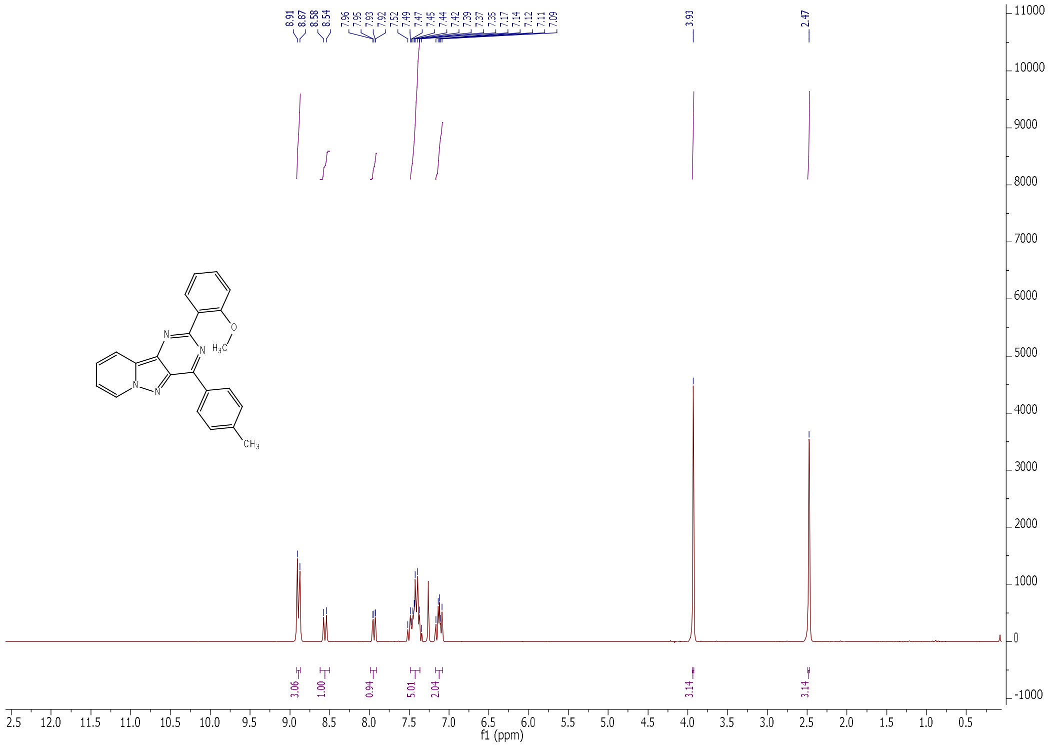

^{13}C NMR (101 MHz, $CDCl_3$)

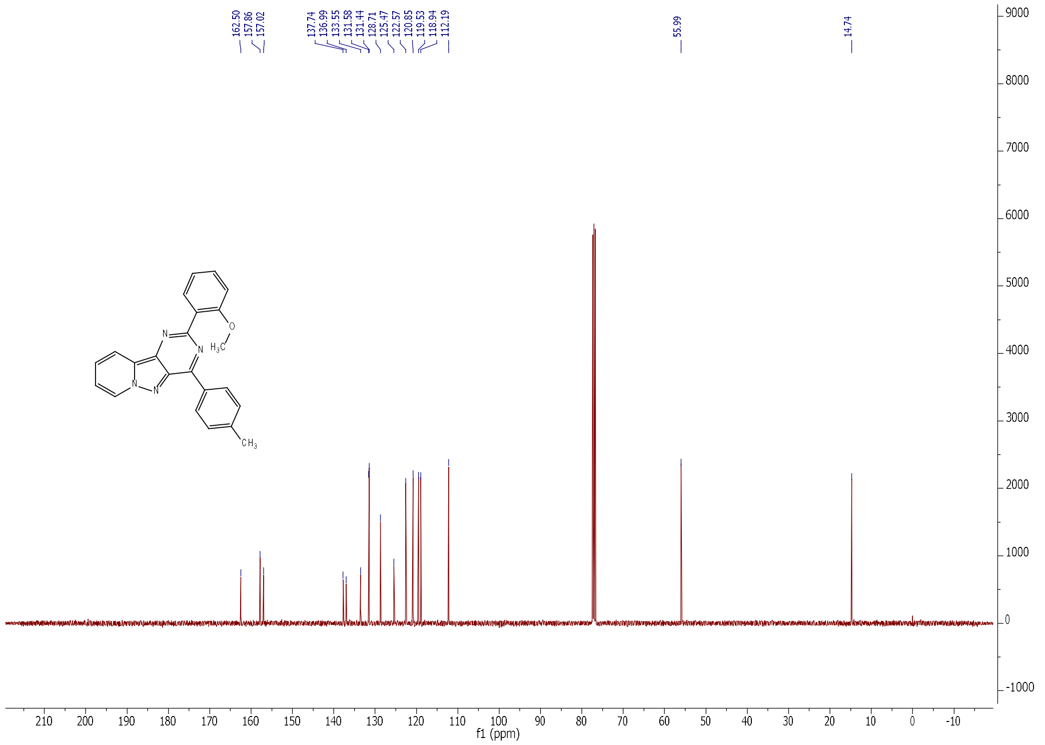

molecules

MDPI

2-(4-fluorophenyl)-4-(p-tolyl)pyrido[3,4]pyrazolo[1,3-b]pyrimidine **(24)**

^{1}H NMR (250 MHz, Chloroform-*d*)

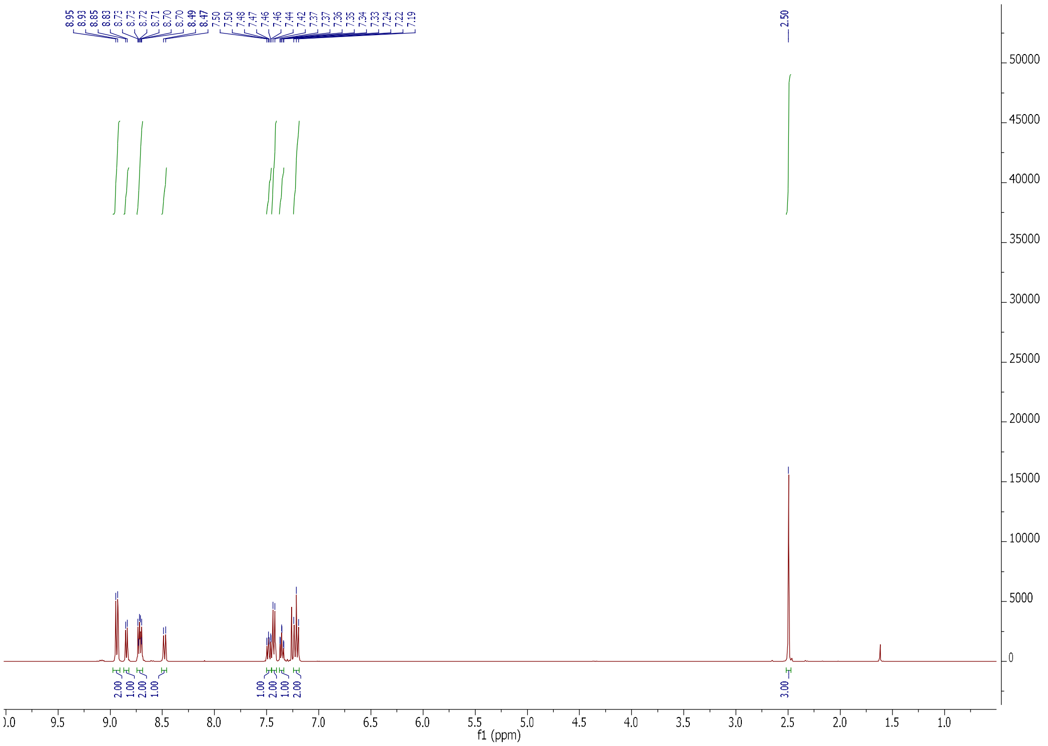

^{13}C NMR (101 MHz, $CDCl_3$)

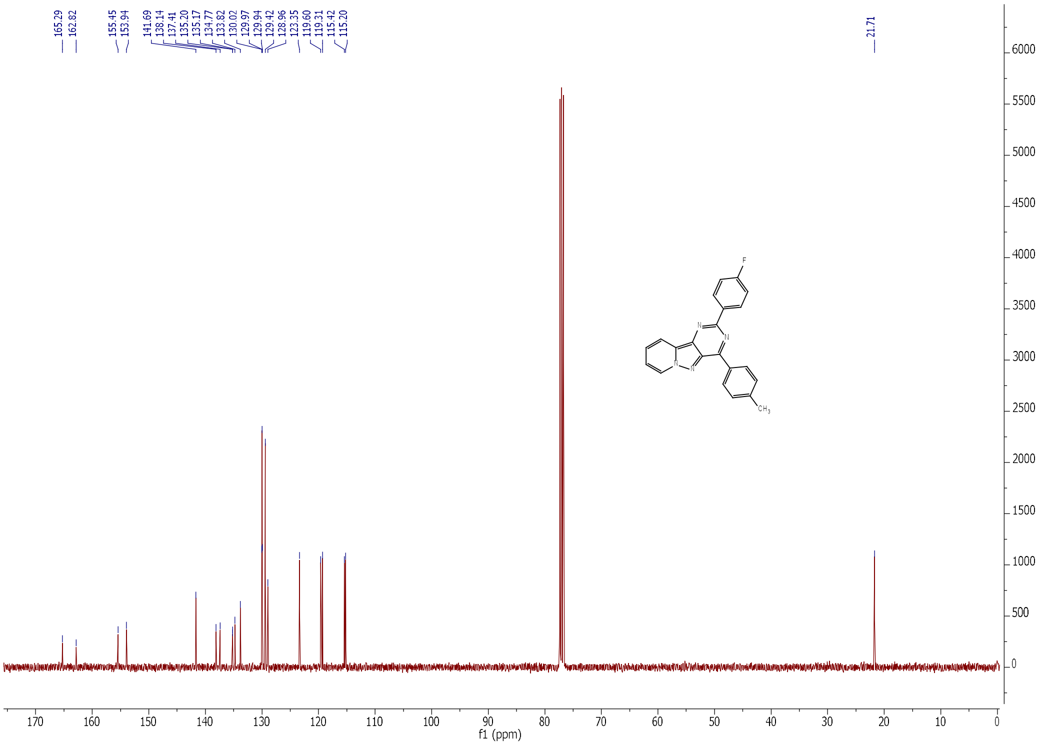

molecules

MDPI

4-(p-tolyl)-2-[4-(trifluoromethyl)phenyl]pyrido[3,4]pyrazolo[1,3-b]pyrimidine **(25)**

^{1}H NMR (400 MHz, Chloroform-*d*)

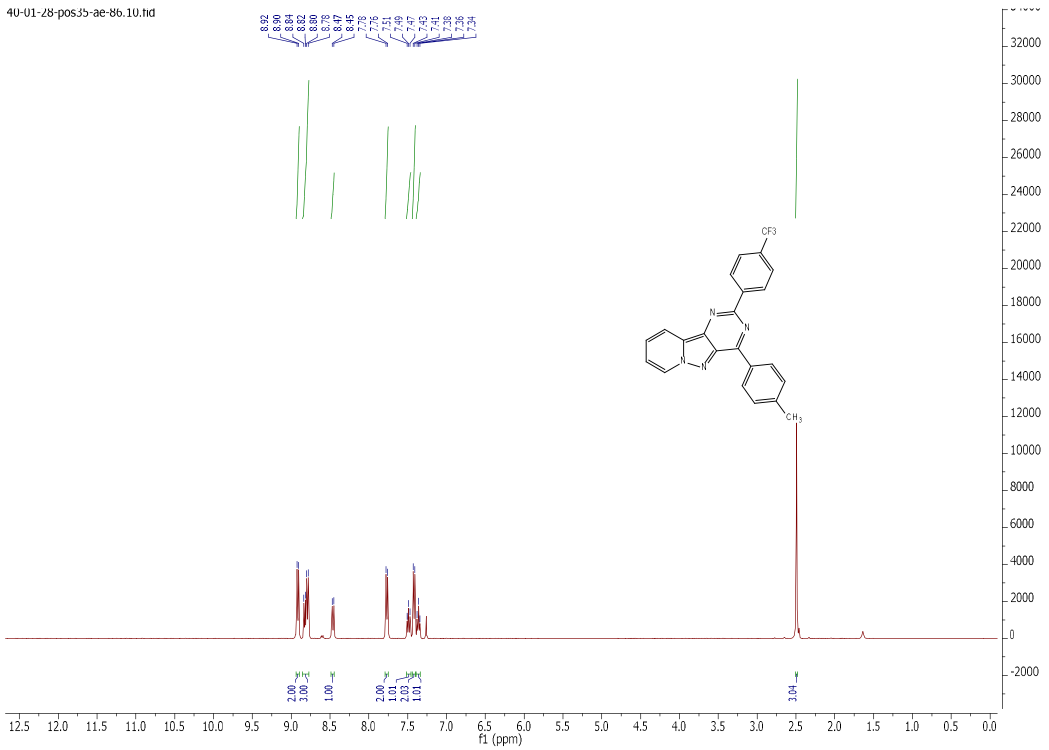

^{13}C NMR (101 MHz, $CDCl_3$)

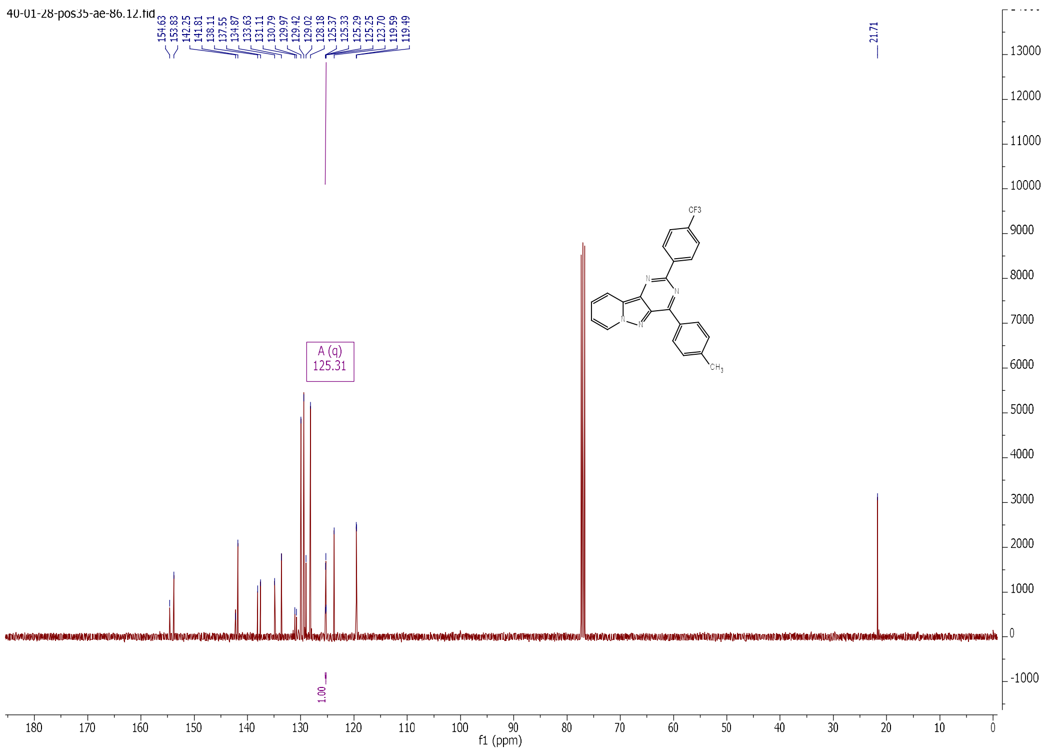

4-[4-(p-tolyl)pyrido[3,4]pyrazolo[1,3-b]pyrimidin-2-yl]phenol **(26)**

^{1}H NMR (250 MHz, Acetone-d_6)

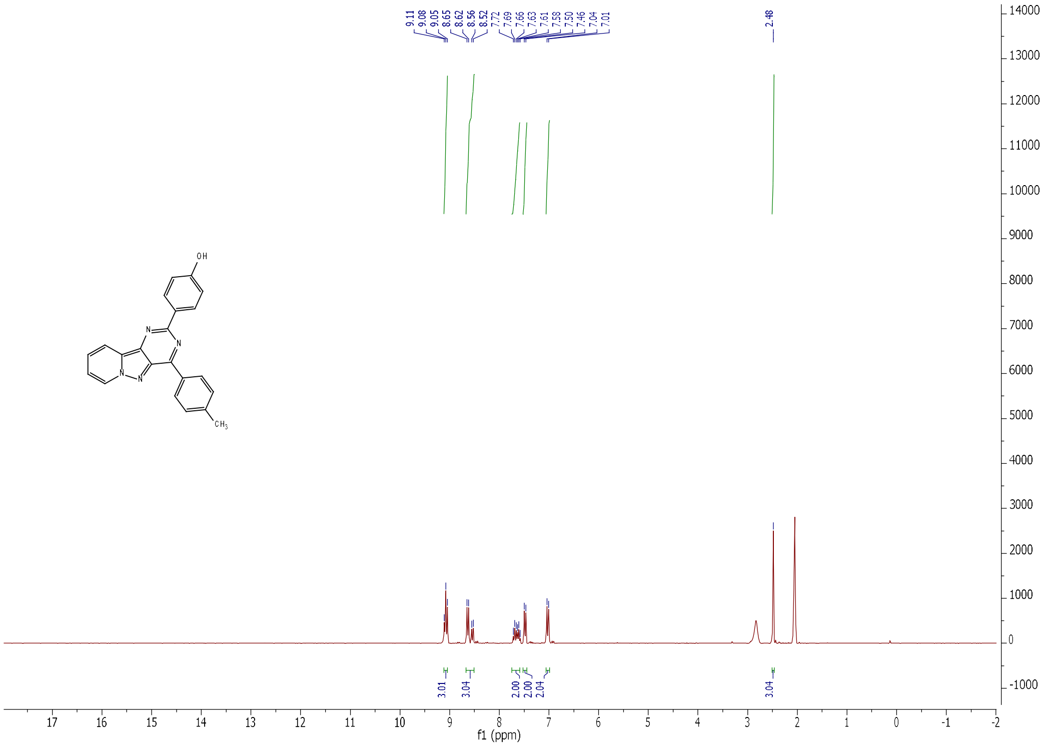

^{13}C NMR (101 MHz, Acetone-d_6).

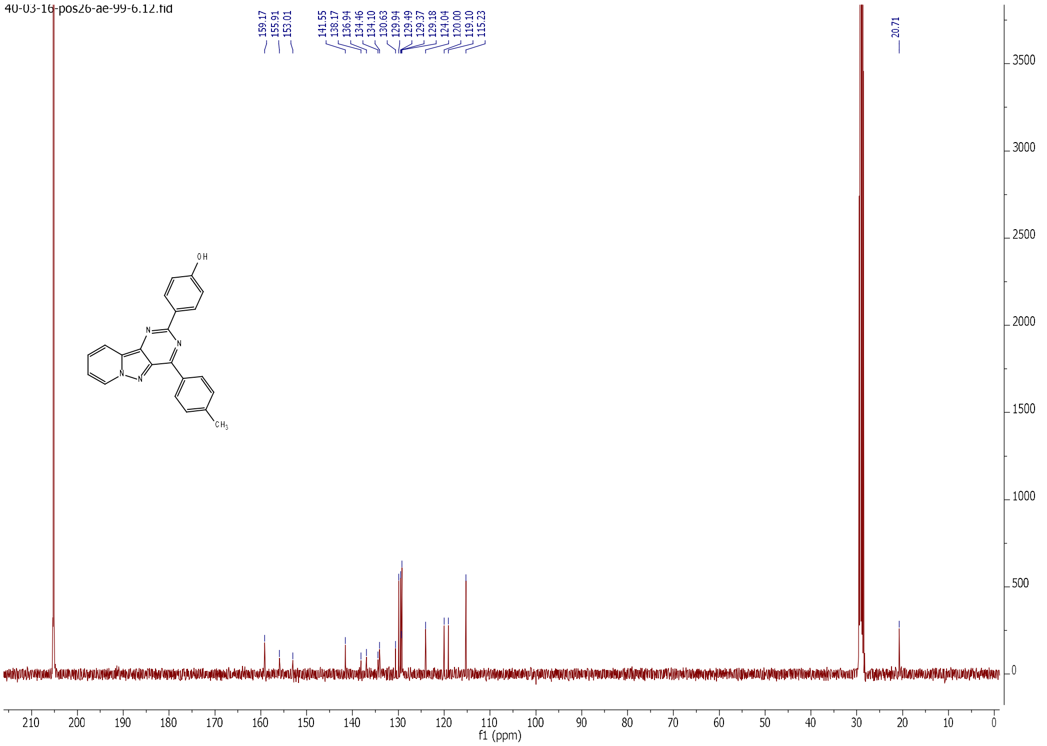

molecules

2-(2-furyl)-4-(p-tolyl)pyrido[3,4]pyrazolo[1,3-b]pyrimidine **(27)**

^{1}H NMR (400 MHz, Chloroform-*d*)

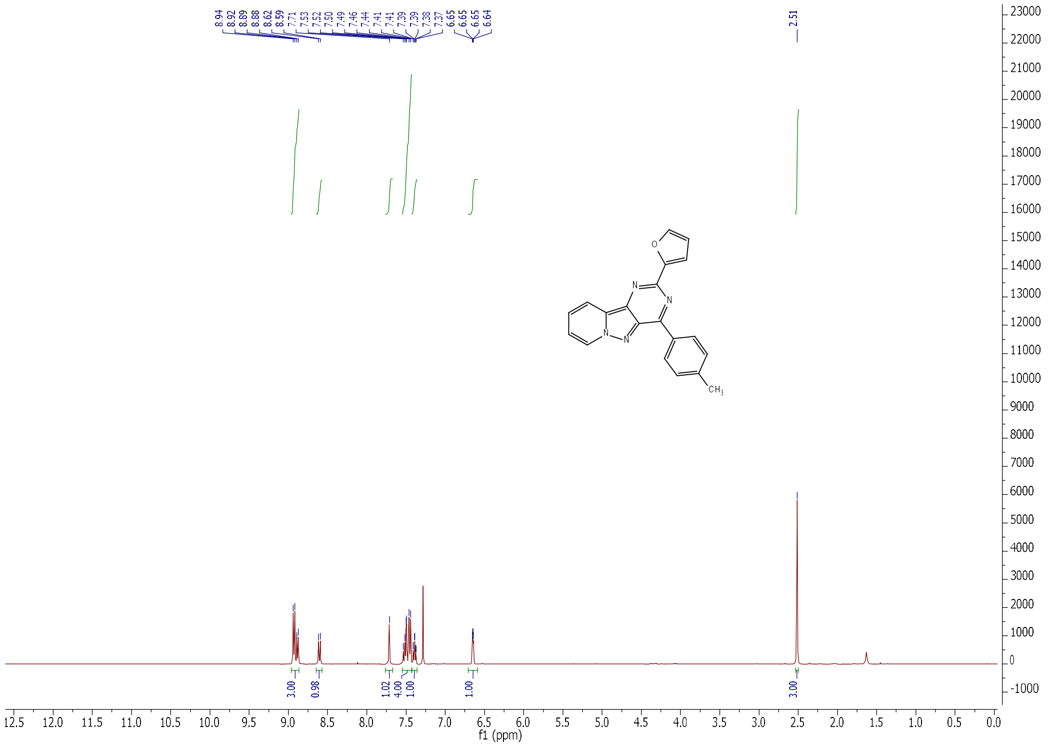

^{13}C NMR (101 MHz, $CDCl_3$)

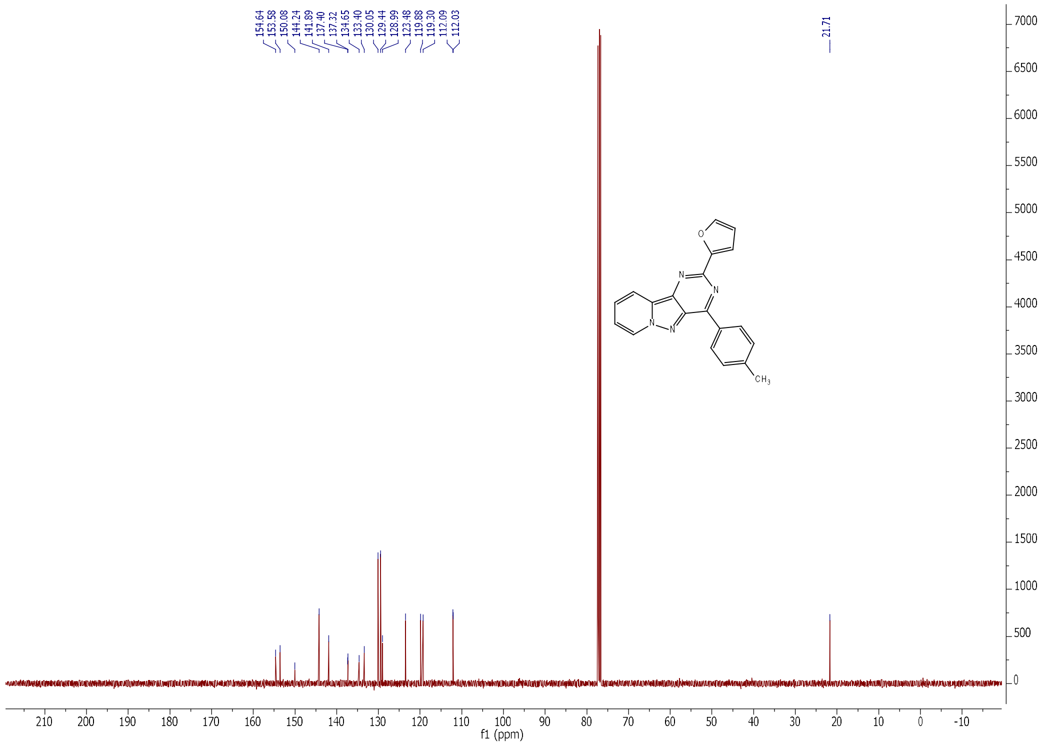

molecules
MDPI

2-(2-tolyl)-4-(p-methoxyphenyl)pyrido[3,4]pyrazolo[1,3-b]pyrimidine **(29)**

^{1}H NMR (400 MHz, Chloroform-*d*)

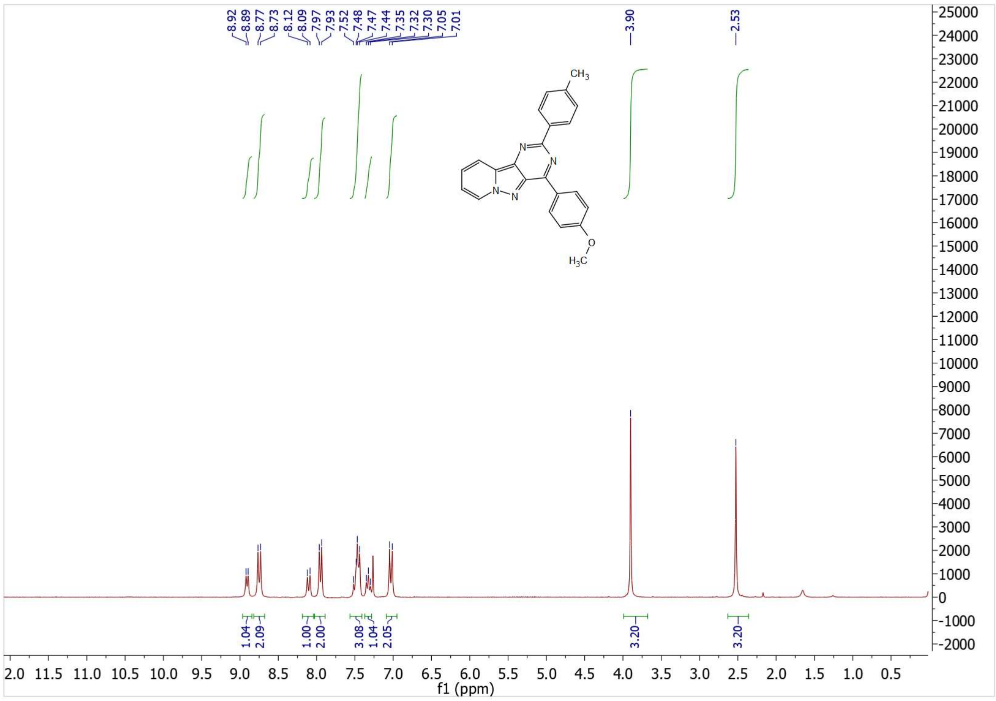

^{13}C NMR (101 MHz, $CDCl_3$)

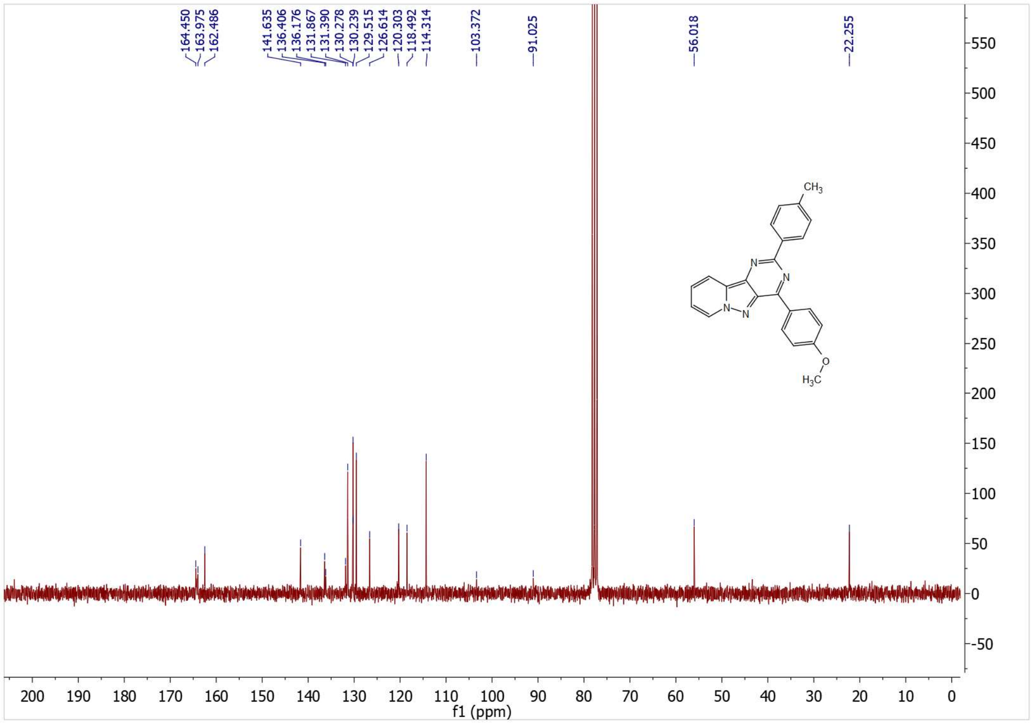

molecules

2-(2-tolyl)-4-(p-fluorophenyl)pyrido[3,4]pyrazolo[1,3-b]pyrimidine **(30)**

^{1}H NMR (400 MHz, Chloroform-*d*)

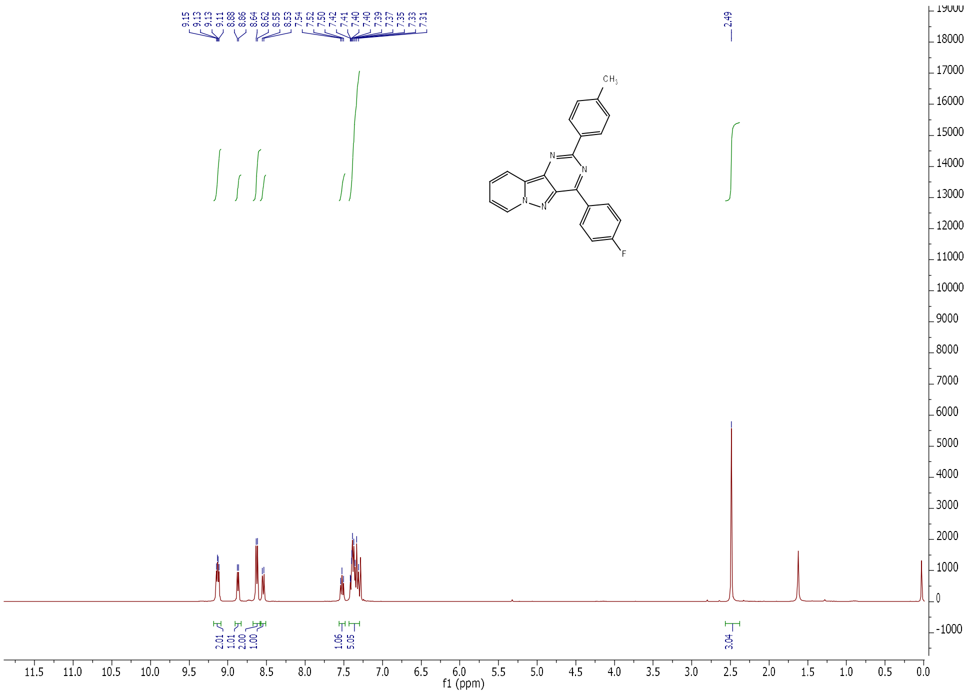

^{13}C NMR (101 MHz, $CDCl_3$)

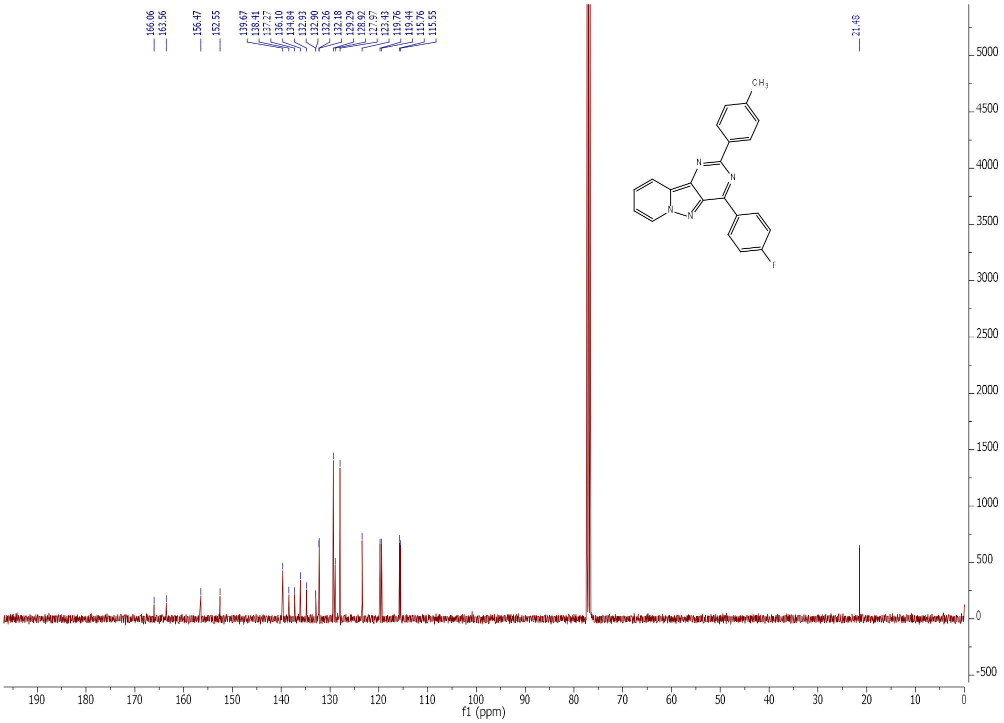

Supplement: Supplementary file 1 [file molecules-23-02740-s001.pdf]
